# Histone H4 deacetylation plays a critical role in early gene silencing during neuronal apoptosis

**DOI:** 10.1186/1471-2202-11-62

**Published:** 2010-05-26

**Authors:** Heather R Pelzel, Cassandra L Schlamp, Robert W Nickells

**Affiliations:** 1 Department of Biomolecular Chemistry, University of Wisconsin, 1300 University Ave, 6671 MSC, Madison, WI, 53706, USA; 2 Department of Ophthalmology and Visual Science, University of Wisconsin, 3310 University Ave, Suite 206, Madison, WI, 53706, USA; 3 Department of Physiology, University of Wisconsin, 1300 University Ave, 125 SMI, Madison, WI 53706, USA

## Abstract

**Background:**

Silencing of normal gene expression occurs early in the apoptosis of neurons, well before the cell is committed to the death pathway, and has been extensively characterized in injured retinal ganglion cells. The causative mechanism of this widespread change in gene expression is unknown. We investigated whether an epigenetic change in active chromatin, specifically histone H4 deacetylation, was an underlying mechanism of gene silencing in apoptotic retinal ganglion cells (RGCs) following an acute injury to the optic nerve.

**Results:**

Histone deacetylase 3 (HDAC3) translocates to the nuclei of dying cells shortly after lesion of the optic nerve and is associated with an increase in nuclear HDAC activity and widespread histone deacetylation. H4 in promoters of representative genes was rapidly and indiscriminately deacetylated, regardless of the gene examined. As apoptosis progressed, H4 of silenced genes remained deacetylated, while H4 of newly activated genes regained, or even increased, its acetylated state. Inhibition of retinal HDAC activity with trichostatin A (TSA) was able to both preserve the expression of a representative RGC-specific gene and attenuate cell loss in response to optic nerve damage.

**Conclusions:**

These data indicate that histone deacetylation plays a central role in transcriptional dysregulation in dying RGCs. The data also suggests that HDAC3, in particular, may feature heavily in apoptotic gene silencing.

## Background

Intrinsic apoptosis in neurons culminates in BAX activation and translocation to the mitochondria, the release of cytochrome c, and the activation of the caspase cascade. BAX translocation marks the committed step of the cell death process [1]. Therefore, investigation of the apoptotic pathway prior to BAX involvement is an important element of developing strategies to intervene in neuronal cell death.

An early event in apoptosis is silencing of normal gene expression. In addition to this, new transcription, required for apoptosis, is activated. This change in transcriptional profile occurs in several models of neurodegeneration, including Huntington's Disease, Alzheimer's Disease, Parkinson's Disease, amyotrophic lateral sclerosis, spinocerebellar ataxia type 3, and the optic neuropathy glaucoma [2-10]. In glaucoma, retinal ganglion cells (RGCs) execute a typical intrinsic apoptotic program. Changes in transcription of several genes in injured RGCs have been shown in experimental glaucoma and after acute injury to the optic nerve. Genes that decrease in expression in RGCs include several that are specifically expressed in these cells, such as *Thy1*, *Brn3b*, *Nrn1*, *Fem1c*, and *Sncg*, [3-5,11-16], as well as several non-cell type specific genes, including *BclX_l_*, *TrkB*, and members of the neurofilament gene family [3,13,14,17]. Of the genes with increased expression, the majority are proapoptotic or stress response genes, such as *Bim*, *cJun*, and several *Hsp*s and caspases [4,5,18-20]. This change in the pattern of gene expression in RGCs occurs before detectable cell loss [4,11,21] and can also be induced by optic nerve crush (ONC) of *Bax *knock-out RGCs, indicating that this event occurs early in the apoptotic pathway. Little investigation has been conducted to understand the mechanism underlying the down-regulation of normal gene expression. The global nature of gene silencing in RGCs, however, suggests that epigenetic changes of the chromatin of actively transcribed genes may be an early step in apoptosis.

Post-translational modifications of histones are well known epigenetic changes that regulate chromatin folding, organization, and gene activity [22]. Histone modifications include phosphorylation, methylation, ubiquitination, and/or acetylation of lysine residues principally in the N-terminal tails [23]. While all of these modifications have an effect on the transcriptional activity, acetylation has the most direct effect [24]. Acetylated histones are typically found in transcriptionally active euchromatic chromatin, whereas transcriptionally inactive heterochromatic chromatin is rich in deacetylated histones. Theoretically, deacetylation is thought to lead to a more compact chromatin structure, which limits access of transcription factors [24]. Alternatively, acetyl groups may facilitate the interactions of chromatin with specific transcription factors containing bromodomains, which recognize and bind acetylated amino acids of other proteins, including histone tails [25]. The acetylation and deacetylation of histones is controlled by opposing protein families called histone acetyltransferases (HATs) and histone deacetylases (HDACs).

Here we show that several RGC specific genes, which decrease in expression after ONC, exhibit a decrease in promoter histone acetylation. This deacetylation is accompanied by an increase in both HDAC2 and HDAC3 expression and the translocation of HDAC3 to the nuclei of dying RGCs. Additionally, inhibition of HDAC activity is able to prevent the ONC-mediated silencing of at least one RGC-specific gene and attenuate the level of RGC death. These results represent one of the first documentations of epigenetic changes associated with neuronal cell death and may provide insight into some of the earliest changes occurring in dying RGCs.

## Results

### Nuclear HDAC activity is increased after ONC

**Figure 1:**
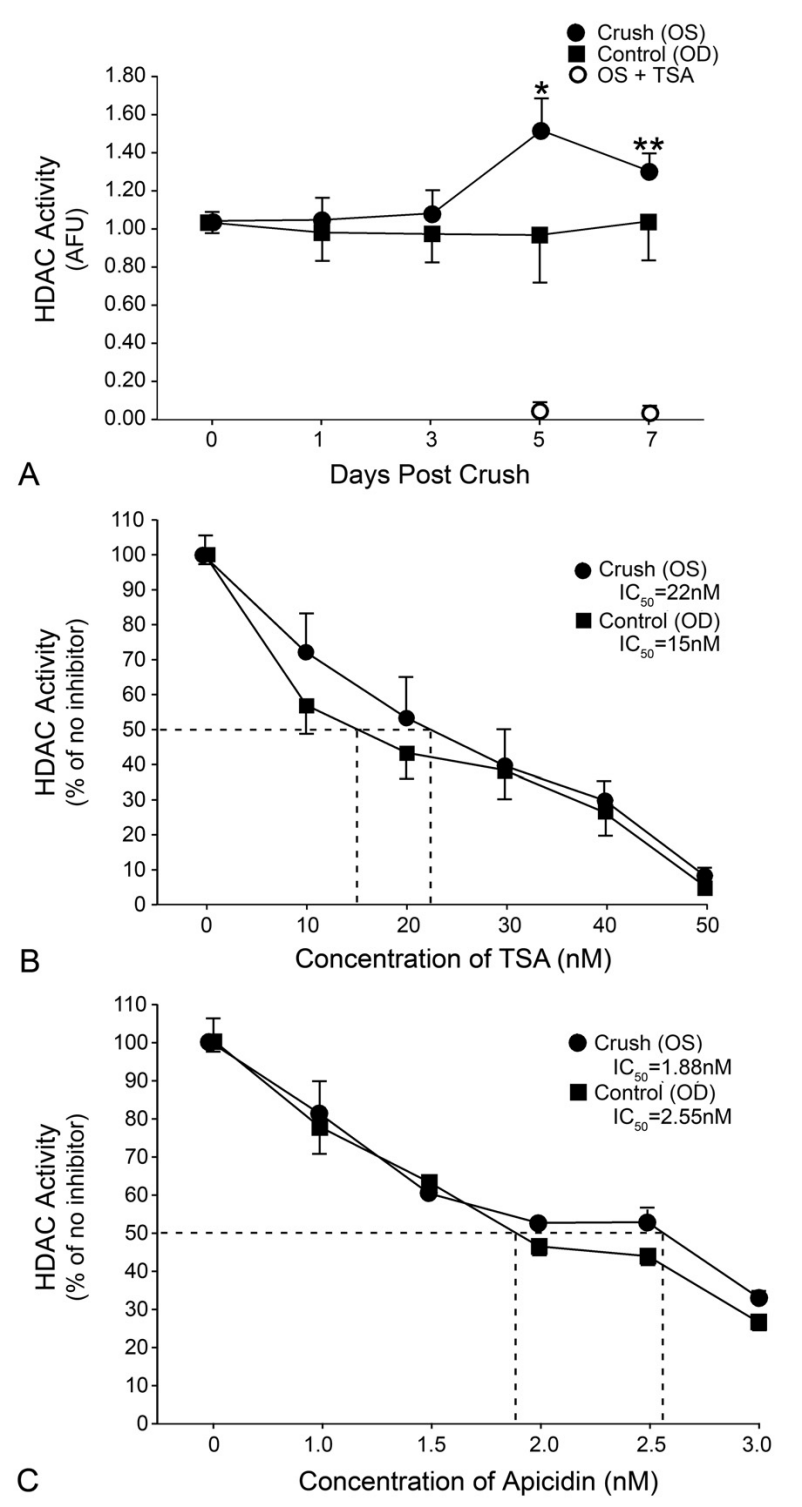
**Nuclear histone deacetylase (HDAC) activity in the retina is increased following optic nerve crush (ONC)**. The data shown was adjusted for protein input and normalized to day 0 control samples. The mean (± SE) HDAC activity is shown (n = 3-5 retinas per data point). (A) No increase in HDAC activity was observed in control eyes (OD) post ONC. There was a significant increase in nuclear HDAC activity in the crush samples (OS) at both 5 and 7 days post ONC (*P = 0.0014 for day 5 vs. day 0 and **P = 0.0006 for day 7 vs. day 0). HDAC activity in this assay was completely inhibited by 500 nM of trichostatin A (TSA). (B) HDAC activity in nuclear extracts from retinas of mice 5 days post ONC was titrated with TSA (n = 3 samples at each concentration). Both control and experimental samples displayed similar inhibition kinetics with an IC_50 _value ranging from 15 -- 22 nM TSA, comparable to the reported IC_50 _values of 1 -- 10 nM for class I HDACs [61]. (C) HDAC activity in the same samples as (B) was titrated with the HDAC2 and HDAC3 selective inhibitor apicidin [62]. Apicidin results in an 80% reduction in nuclear HDAC activity, yielding an IC_50 _ranging from 1.88 to 2.55 nM, consistent with the reported HDAC3 IC_50 _for apicidin [26]. These data suggest that the majority of nuclear HDAC activity in the retina is provided by class I HDACs, principally HDAC2 and HDAC3. AFU, arbitrary fluorescence units.

Nuclear HDAC activity was measured in retinal nuclear protein extracts isolated at 1, 3, 5, and 7 days following ONC (Figure 1A). No significant change was detected in fellow control eyes (OD). Experimental eyes (OS) exhibited an ~50% increase in activity at day 5 post ONC (P = 0.0014). Activity was also significantly higher than control eyes at 7 days post ONC (P = 0.0006), although lower than day 5 experimental eyes. Nuclear HDAC activity in experimental and control eyes was completely inhibited by trichostatin A (TSA), indicating the presence of predominantly class I and II HDACs in this fraction (Figure 1A).

Dose-dependent inhibition of nuclear HDAC activity was also performed with different inhibitors to help evaluate which HDACs were active both before and after ONC. TSA-mediated inhibition of control and crush nuclear extracts from day 5 retinas showed a dose-dependent decrease in HDAC activity with an IC_50 _ranging from 15 to 22 nM (Figure 1B). A similar loss of activity was observed in extracts treated with the selective class I inhibitor, valproic acid (data not shown). To further refine which class I HDACs may be contributing to the retinal nuclear activity, we repeated this experiment using apicidin, which is selective for HDACs 2 and 3. HDAC activity was also nearly completely inhibited with apicidin, yielding an IC_50 _ranging from 1.88 to 2.55 nM (Figure 1C). These values agreed with the reported IC_50 _value for HDAC3 (2.5 nM) using this inhibitor [26] and suggest that HDACs 2 and 3 contribute the majority of nuclear HDAC activity in the retina.

**Figure 2:**
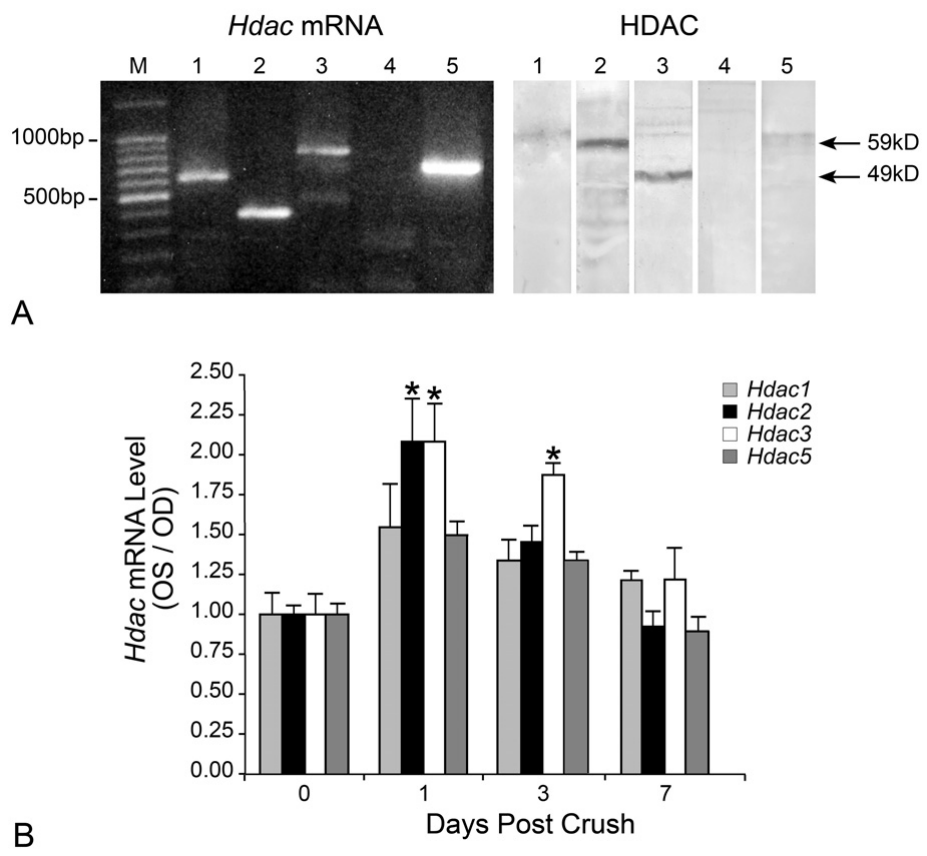
**HDACs 2 and 3 are prominently expressed in the mouse retina and increase in expression following optic nerve injury**. (A) The presence of transcript and protein of several HDAC isoforms was characterized for the murine retina. HDACs 1-5 were examined as they have all been shown to deacetylate nuclear histones. *Left panel*, non-quantitative RT-PCR of control retina cDNA generated cDNA amplimers corresponding to *Hdacs *1-3 and 5, but not *Hdac4*. The identity of each amplimer was verified by sequencing. *Right panel*, HDAC proteins were identified in control retina lysates by western blot analysis using antibodies against the indicated HDAC isoforms. Bands of the reported nuclear weights corresponding to HDACs 1-3 and 5 were detected. Consistent with the RT-PCR experiments, no band was detected for HDAC4. (B) Changes in HDAC transcript abundance after ONC were analyzed by qPCR (n = 8-10 retinas per time point). Transcript levels were expressed as the ratio of crush (OS): control (OD) retinas, and this ratio was normalized to day 0 samples. There were modest, but significant increases in *Hdac*s 2 and 3 at 1day post crush (*P = 0.0098 and P = 0.0089, respectively) and this increase persisted to 3 days post crush for *Hdac3 *(*P = 0.0024).

In addition to measuring nuclear HDAC activity, we also characterized which HDACs were expressed in the mouse retina by both mRNA and protein analysis. HDACs 1-5 were selectively examined because they have all been reported to be active in nuclei and are known to affect histone acetylation levels [27]. By RT-PCR and western blot analysis, HDACs 1, 2, 3, and 5, but not HDAC4 were detected in normal retinas (Figure 2A). At the protein level, bands of 59 kD and 49 kD, corresponding to HDACs 2 and 3, respectively, appeared to be most abundant. This is similar to a previous report of the pattern of HDACs expressed in normal brain tissue [27]. To examine the change in HDAC expression in response to ONC, quantitative RT-PCR (qPCR) analysis was conducted on samples isolated from several time points following surgery (Figure 2B). By 1 day post ONC, the mRNAs for *Hdacs *2 and 3 doubled (P = 0.0098 and P = 0.0089, respectively), while transcripts for *Hdacs *1 and 5 showed modest, but not significant increases. At 3 days post ONC, only *Hdac3 *transcripts remained significantly elevated (P = 0.0024). By 7 days post ONC, no significant elevation of any of the examined *Hdacs *was detected.

### HDACs 2 and 3 in injured retinas

**Figure 3:**
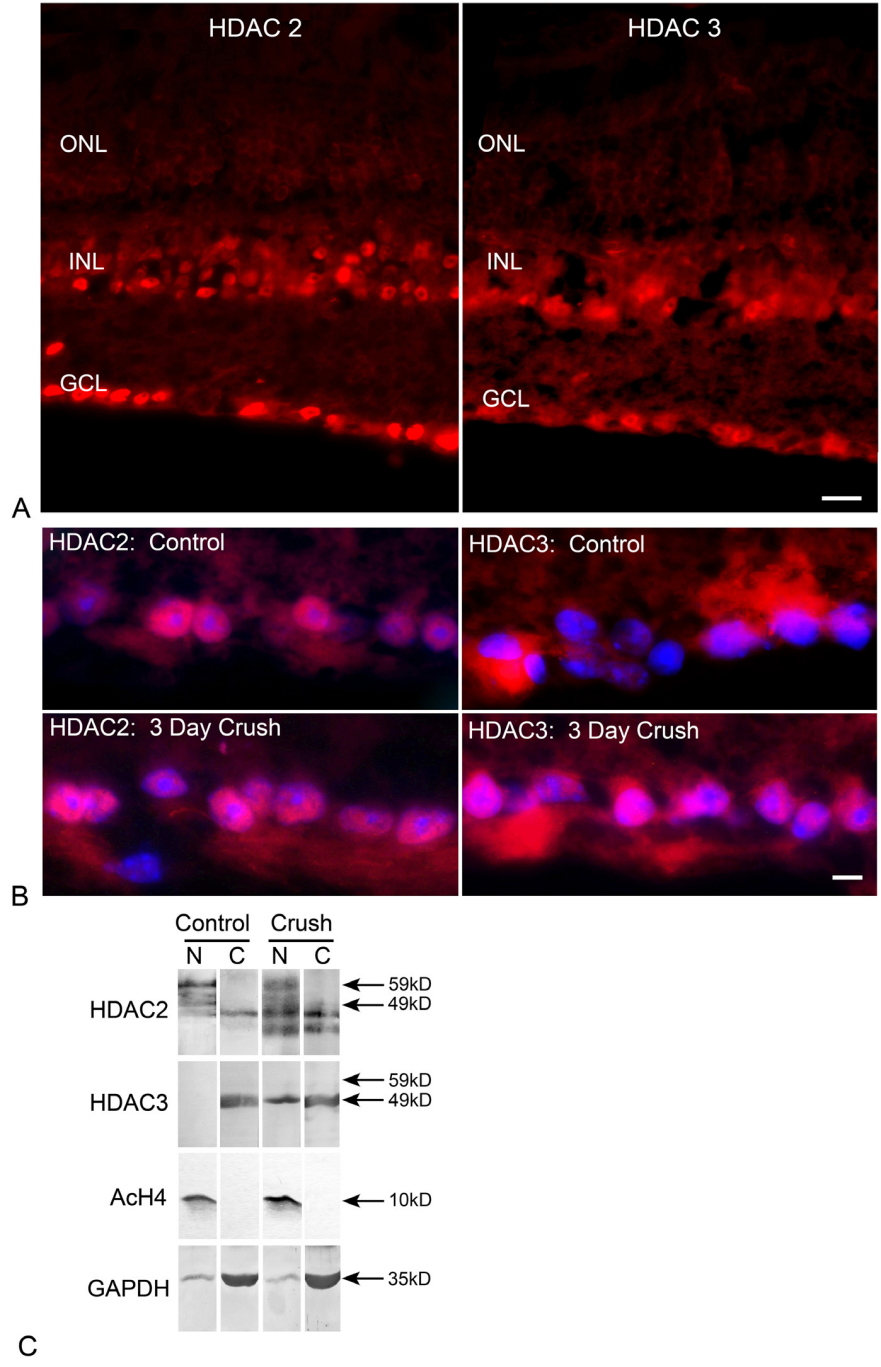
**HDAC2 and HDAC3 are expressed by cells in the ganglion cell layer (GCL)**. (A) Nuclei in the GCL and inner nuclear layer (INL) were strongly labeled for HDAC2. HDAC3 labeling was more diffuse, but also labeled cells in the GCL and INL. Scale bar = 25 μm. (B) Sections from control retinas and retinas 3 days after optic nerve crush. High-magnification images of the GCL are shown. Sections were counter-stained with DAPI to highlight nuclei. In control retinas, HDAC2 was localized to nuclei of GCL cells. HDAC3 labeling was principally in the cytoplasm of GCL cells, although some label appeared to be distributed in or around cellular nuclei. In the GCL of crush retinas, HDAC2 remained nuclear (lower left), while HDAC3 labeling was clearly detected in the nuclei of some cells. Scale bar = 5 μm. Photomicrographs of the individual channels of these merged images are shown in additional file 1. (C) Western blot analysis of HDACs 2 and 3 in cytoplasmic (C) and nuclear (N) fractions of control and experimental retinas 3 days after optic nerve crush. A 59 kD band corresponding to HDAC2 was exclusive to the nuclear fractions in both control and crush retinas. The 49 kD band of HDAC3 was present only in the cytoplasmic fraction in the control eyes, but was distributed between both nuclear and cytoplasmic fractions in the crush samples suggesting nuclear translocation of HDAC3 after ONC. Control antibodies for AcH4 (nuclear) and GAPDH (cytoplasmic) are shown to monitor the relative purity of each fraction.

Since both *Hdac *2 and 3 transcripts showed a significant increase following ONC and the majority of HDAC activity was sensitive to apicidin, we characterized these HDACs in the retina before and after ONC. To determine the localization of these isoforms, immunofluorescence was performed on retinal cryosections from control and crush eyes. Both HDAC2 and HDAC3 were present in cells of the ganglion cell layer (GCL) and inner nuclear layer (INL) of control eyes (Figure 3A). At higher magnification of the GCL of control retinas (Figure 3B, top panels), HDAC2 colocalized with 4,6-diamindino-2-phenylindole (DAPI), indicating that it was nuclear, consistent with reports that this is an exclusively nuclear protein [27]. In contrast, HDAC3 labeling was diffuse and appeared to be predominantly cytoplasmic with minimal overlap with DAPI labeling. This was also in agreement with previous reports that HDAC3 contains both a nuclear localization signal and a nuclear export signal [27], and can exist in both the cytoplasm and nuclei of cells [28].

Because subcellular localization of HDACs is a common mechanism of controlling HDAC activity [29], we also examined the distribution of HDAC2 and HDAC3 after ONC. In sections from retinas 3 days post ONC (Figure 3B, lower panels), HDAC2 remained localized to nuclei of the GCL. HDAC3 localization in the GCL, however, appeared to change after ONC, with cells showing both cytoplasmic and nuclear localization, as determined by colabeling with DAPI staining (Figure 3B and additional file 1: Localization of HDACs 2 and 3 before and after optic nerve crush). To confirm these findings, western blot analysis was conducted on nuclear and cytoplasmic fractions isolated from control and crush retinas. As shown in Figure 3C, a band at 59 kD, corresponding to HDAC2, was present in the nuclear fractions of both control and ONC retinas. A 49 kD band, corresponding to HDAC3, was present in the cytoplasmic fraction from control retinas, but was both cytoplasmic and nuclear in the experimental crush retinas consistent with the nuclear translocation of this protein after ONC. As a control for the fractionation, the blots were also probed for acetylated histone H4 and GAPDH as nuclear and cytoplasmic controls, respectively.

### Histone H4 acetylation in the GCL decreases following ONC

**Figure 4:**
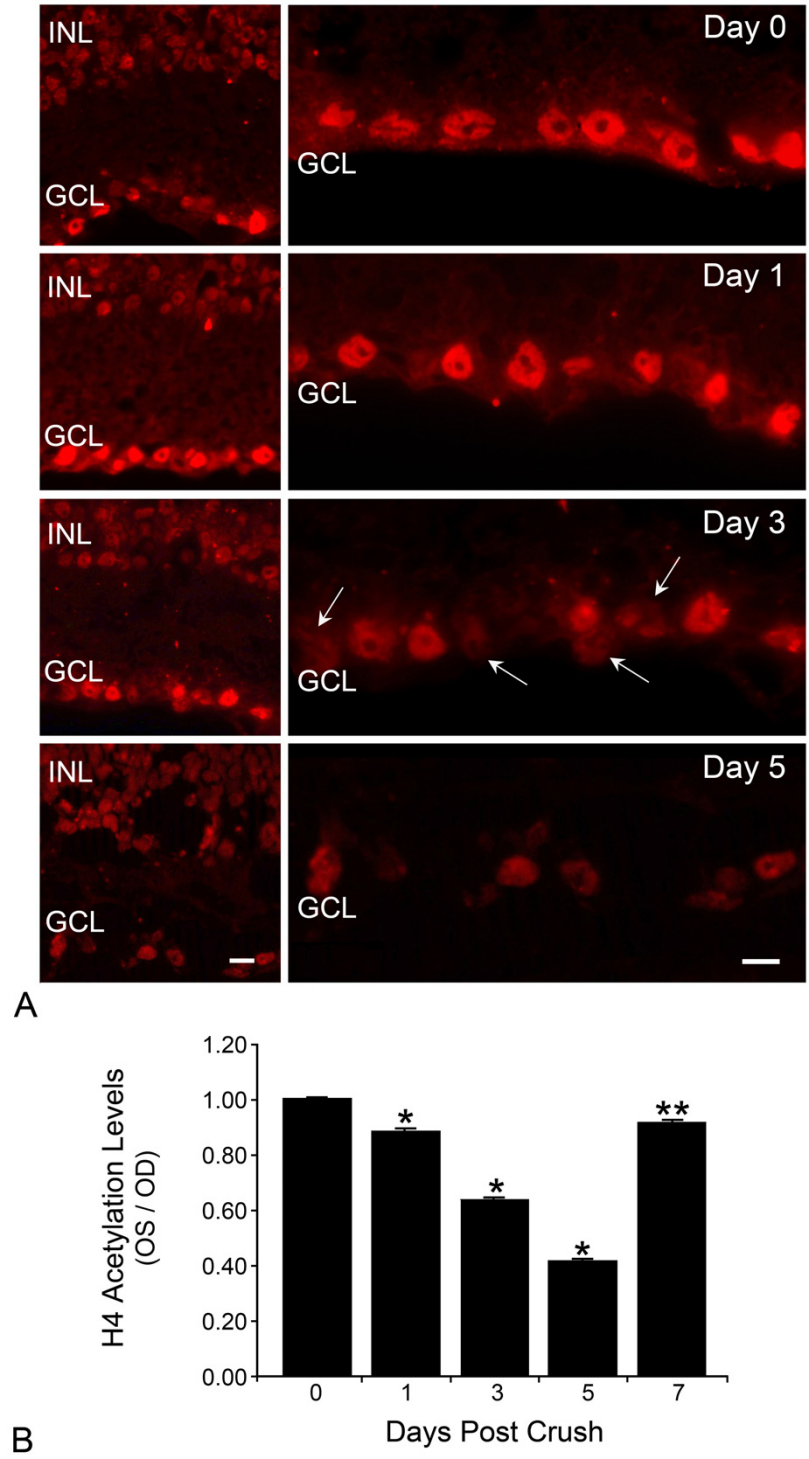
**Acetylated histone H4 is depleted in the ganglion cell layer (GCL) nuclei following optic nerve crush (ONC)**. (A) *Left panels*, sections from a control retina and retinas after ONC were labeled for acetylated histone H4 (AcH4). Nuclei in the outer nuclear layer (not shown), inner nuclear layer (INL) and GCL of the retina were labeled. AcH4 labeling in the INL remained constant at all time points examined. Labeling in the GCL progressively decreased after ONC. Scale bar = 20 μm. *Right panels*, Higher magnification of cells of the GCL after ONC. All images were taken at a fixed exposure time. At 1 day post ONC, AcH4 labeling was slightly decreased. At 3 days post ONC, several nuclei showed pronounced loss of label (arrows). At 5 days post ONC, only a subset of cells in the GCL remained labeled for AcH4. Scale bar = 10 μm. (B) Quantification of AcH4 staining intensity of cells in the GCL. Staining intensity was normalized to AcH4 staining in cells of the INL for each section examined. Results are shown as the ratio of staining intensity between experimental (OS) and control (OD) eyes of the same animals (n < 99 cells counted per layer). AcH4 immunoreactivity significantly decreased during the first 5 days post ONC (*P ≤ 0.0001 OS vs. OD). By 7 days post ONC, AcH4 staining appeared to recover, but was still significantly lower than control retinas (**P = 0.041 OS vs. OD). Staining recovery may reflect an increase in the proportion of unaffected GCL amacrine cells as ganglion cells die.

Histones H3 and H4 are substrates of HDACs 2 and 3. In particular, deacetylation of at least 5 different lysine residues of H4 have been implicated in transcriptional silencing [24]. To determine if there was a decrease in acetylation of histones in injured RGCs, concomitant with the increase in HDAC activity and increased nuclear presence, we examined retinal cryosections taken from control and experimental retinas with a polyclonal antibody against pan-acetylated histone H4 (AcH4). Figure 4A shows low magnification images of the INL and GCL (left panels), and higher magnification images of just the GCL (right panels). Nuclear labeling was detected in both the INL and GCL of the control retinas (top panels). After ONC, the label intensity remained consistent in the INL, but there was an apparent and progressive decrease in labeling of the GCL. High magnification images (Figure 4A, right panels) indicated that the decrease in label intensity was not only due to a loss of cells in this layer, but also to a decrease in the labeling of individual cells within this layer. To verify the decrease in label intensity of individual nuclei in the GCL, fluorescent pixel intensity of cells in this layer were measured and normalized to pixel intensity of AcH4 labeling in the INL of the same section. Compared to control eyes, the average AcH4 label intensity of GCL nuclei in experimental retinas progressively decreased to ~45% by 5 days post ONC (Figure 4B, P < 0.0001 for days 1, 3, and 5 vs. control retinas). By 7 days post ONC, many RGCs are in the later stages of apoptosis [30]. Consistent with this, we observed an actual increase in the average AcH4 label intensity of cells remaining in this layer at 7 days. Although this average was still significantly below control levels (P = 0.04), it may reflect that unaffected amacrine cells made up a larger proportion of nuclei assayed at this time point.

### Characterization of HDAC3 translocation and deacetylation of histone H4 in dying cells

**Figure 5:**
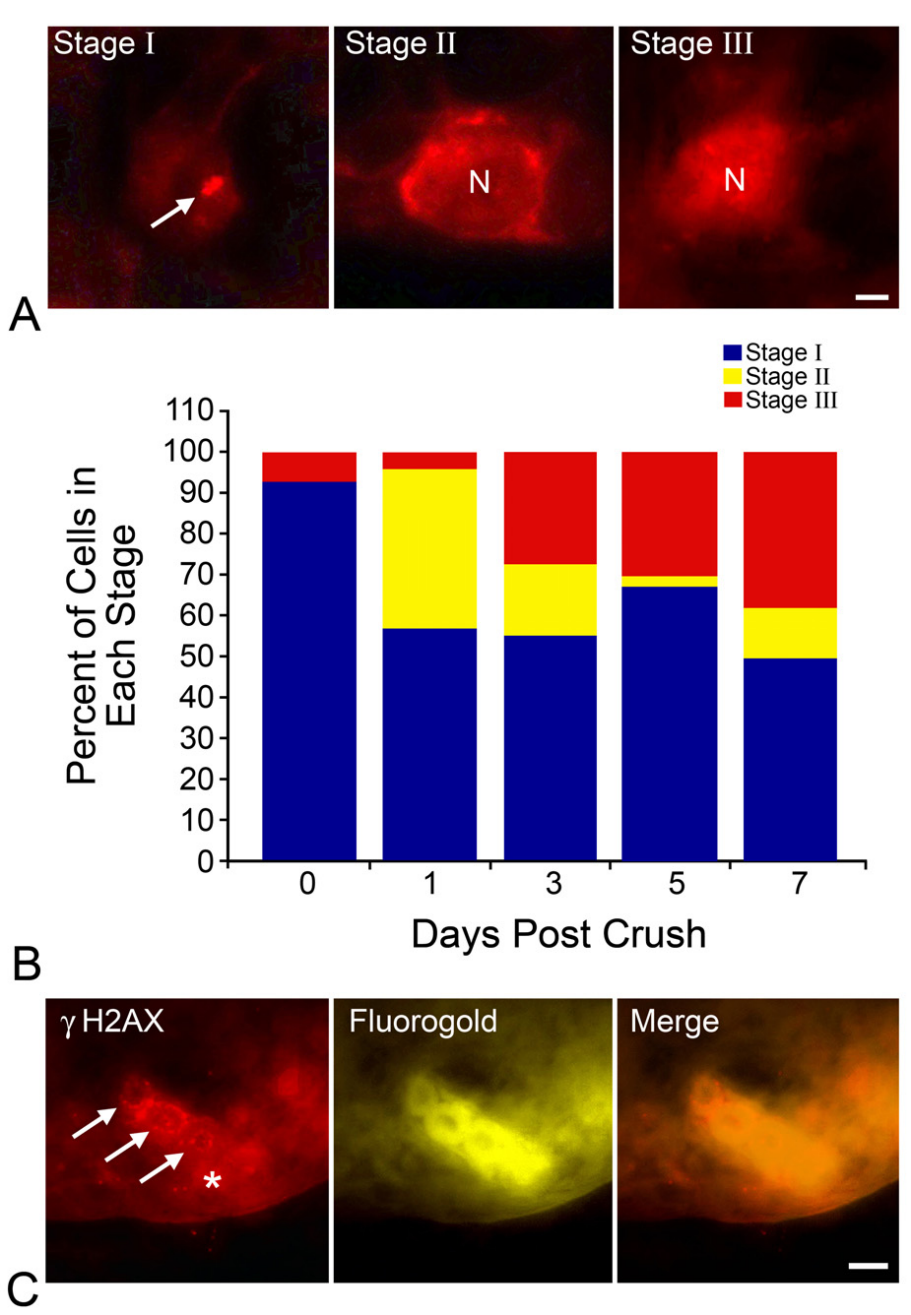
**Localization of γH2AX in apoptotic retinal ganglion cells (RGCs)**. Three stages of γH2AX localization in the ganglion cell layer (GCL) were apparent. (A) γH2AX labeling in healthy cells (Stage I) was detectable as a prominent spot associated with the nucleolus (arrow). Stage II labeling was characterized by a cytoplasmic perinuclear ring. Strong nuclear labeling (N) was indicative of stage III γH2AX localization. Scale bar = 3 μm. (B) Quantification of γH2AX labeling in control and crush retinas. In control eyes (day 0), the majority of cells in the GCL exhibited stage I labeling with a small number of cells showing stage III labeling. As early as 1 day post ONC, stage II labeling was present in ~40% of cells in the GCL with a small number of stage III labeled cells. Stage III labeling increased through days 3, 5, and 7 post ONC. No more than 50% of cells in the GCL exhibited stage II or III γH2AX labeling, suggesting that a subset of cells were affected. (C) To verify that the subset of γH2AX labeled cells were RGCs, they were prelabeled with Fluorogold (FG) prior to ONC and staining for γH2AX (5 days post ONC). The images show 3 FG positive cells in the GCL with stage II γH2AX labeling (arrows), while a fourth FG labeled cell (asterisk) shows relatively weak γH2AX labeling. In the control eyes, < 15% of FG-positive RGCs exhibited stage II or III γH2AX labeling versus 85% of FG-positive RGCs in experimental eyes. Scale bar = 10 μm.

The increase in nuclear HDAC3 localization and an apparent decrease in histone H4 acetylation in some cells of the GCL following ONC is consistent with the concept of widespread histone deacetylation taking place in dying RGCs, which are the principal cell type affected by the crush procedure. Importantly, we wanted to verify that these changes were characteristic of dying cells. To address this, we first identified dying cells in retinal cryosections with an antibody against phosphorylated H2AX (γH2AX). The phosphorylation of the histone H2A variant, H2AX, is a known marker of apoptosis, and its appearance coincides with early onset DNA damage that precedes mitochondrial involvement in the cell death program [31]. In the GCL of injured retinas, γH2AX underwent a progressive change in localization that allowed us to group the cells into three stages of γH2AX labeling (Figure 5A). Stage I labeling was principally found in control retinas and in some GCL cells in injured retinas and was characterized by little to no γH2AX labeling except for a densely labeled spot associated with the nucleoli. Stage II was distinguishable by strong perinuclear labeling, while stage III had strong nuclear labeling. Stage II labeled cells began to appear in the injured GCL as early as 1 day post ONC. Progressively after this, an increasing proportion of cells exhibited stage III labeling. By 5-7 days post ONC, 50-60% of the cells in this layer were either stage II or stage III for γH2AX, consistent with estimates that RGCs make up 60% of the cells in the GCL (Figure 5B) [32,33]. To verify labeling in RGCs, we also retrogradely labeled them with fluorogold prior to ONC. After crush surgery, an estimated 85% of the fluorogold positive cells exhibited stage II or stage III γH2AX labeling (Figure 5C).

**Figure 6:**
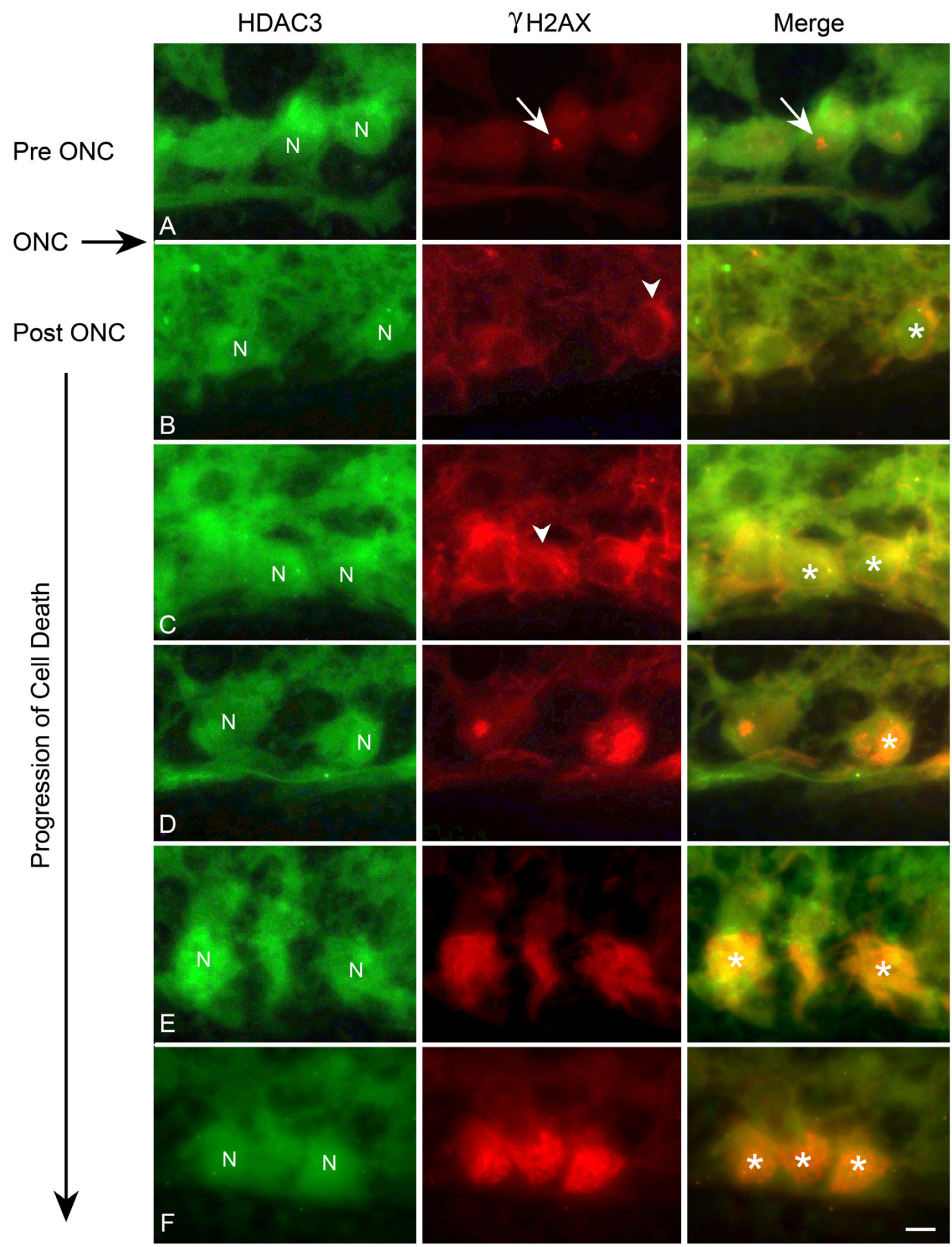
**HDAC3 translocates to the nuclei of apoptotic cells**. Retinal sections from eyes before optic nerve crush (ONC) and at several points after ONC were double labeled with antibodies against HDAC3 (green) and γH2AX (red), to identify apoptotic cells (see Figure 5). (A) Cells in the ganglion cell layer prior to ONC exhibited diffuse cytoplasmic HDAC3 labeling and a densely labeled spot of γH2AX (stage I labeling), which associated with the nucleoli (arrows). Representative unlabeled nuclei are indicated (N). (B) Following ONC, HDAC3 labeling began to appear in the nuclei (asterisk in merged image), while γH2AX labeling was detectable as a cytoplasmic perinuclear ring (stage II labeling, arrowhead). (C) HDAC3 labeling continued to increase in the nuclei (asterisks), while the overall intensity of γH2AX labeling increased in both the cytoplasm (arrowhead) and the nuclei, which appear yellow in the merged image (asterisks). (D) At later time points post ONC, a greater proportion of cells exhibited nuclear colocalization of HDAC3 and γH2AX (stage III labeling, arrowhead). (E, F) HDAC3 and γH2AX labeling continued to colocalize (asterisks), although nuclei of dying cells were less well defined. Scale bar = 5 μm.

Figure 6 illustrates the change in HDAC3 and γH2AX labeling in the retina as RGCs progressed through apoptosis. HDAC3 appeared to be cytoplasmic in control cells (Figure 6A), and shortly after ONC, was found co-localized with stage II γH2AX labeled cells in the cytoplasm (Figure 6B). In other cells, however, HDAC3 staining was present in the nuclei, while γH2AX staining was still localized as a perinuclear ring in the cytoplasm (Figure 6C). In mid-to late stages post ONC, double-labeled cells in the GCL exhibited stage III γH2AX labeling and nuclear localization of HDAC3 (Figure 6D-F).

**Figure 7:**
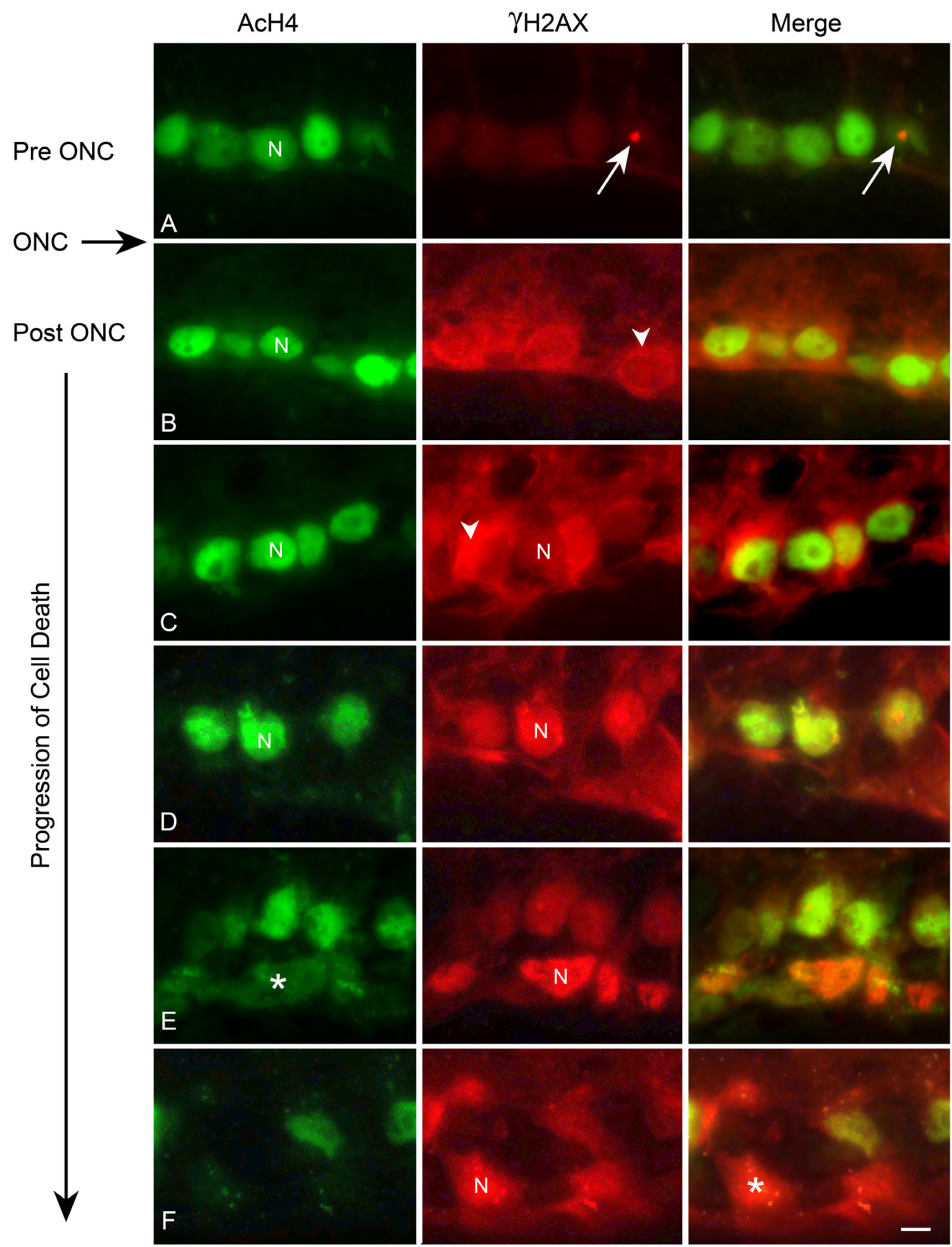
**Widespread deacetylation of histone H4 occurs in apoptotic cells**. Retinal sections from eyes before optic nerve crush (ONC) and at several points after ONC were double labeled with antibodies against acetylated histone H4 (AcH4, green) and γH2AX (red), to identify apoptotic cells (see Figure 5). (A) Control ganglion cell layer nuclei (N) were strongly labeled for AcH4. Arrows indicate focal γH2AX labeling associated with nucleoli (stage I). (B) Shortly after ONC, γH2AX labeling became more apparent as a perinuclear ring, principally localized to the cytoplasm (arrowhead, stage II), while nuclei of these cells still exhibited strong labeling for AcH4. (C, D, E) At subsequent time points after ONC, γH2AX labeling in nuclei (N) progressively increased (stage III), while nuclear AcH4 labeling decreased (asterisk in E). Note a change from green to yellow to red nuclear color in the merged panels. (F) By late in the cell death process, nuclei (N) intensely labeled for γH2AX exhibited little to no immunoreactivity to AcH4 (asterisk). Scale bar = 5 μm. The decrease in AcH4 labeling, and the presence of γH2AX stage III labeling, also correspond to increased DNA fragmentation as assessed by DAPI staining (See additional file 2).

Because of the strictly nuclear presence of AcH4, the colocalization of AcH4 and γH2AX was much different than that observed for HDAC3. In control eyes, the nuclei were strongly labeled for AcH4 and γH2AX was only present as a spot that was associated with the nucleoli (Stage I, Figure 7A). Early after ONC, RGCs with stage II γH2AX localization also presented with strong nuclear labeling with AcH4 (Figure 7B, C). In stage III labeled cells, γH2AX and AcH4 colocalization was initially yellow in merged images, but progressed to orange, then red, indicative of both an increase in γH2AX intensity and a decrease in AcH4 labeling (Figure 7D-F). The nuclear localization of this co-labeling was confirmed with DAPI staining, which also indicated that cells with nuclear γH2AX and decreased AcH4 staining exhibited fragmented and condensed nuclei typical of apoptotic cells (See additional file 2: The deacetylation of histone H4 occurs in cells with nuclear γH2AX and DNA fragmentation).

### Promoter deacetylation is associated with a downregulation of gene expression in injured RGCs

**Figure 8:**
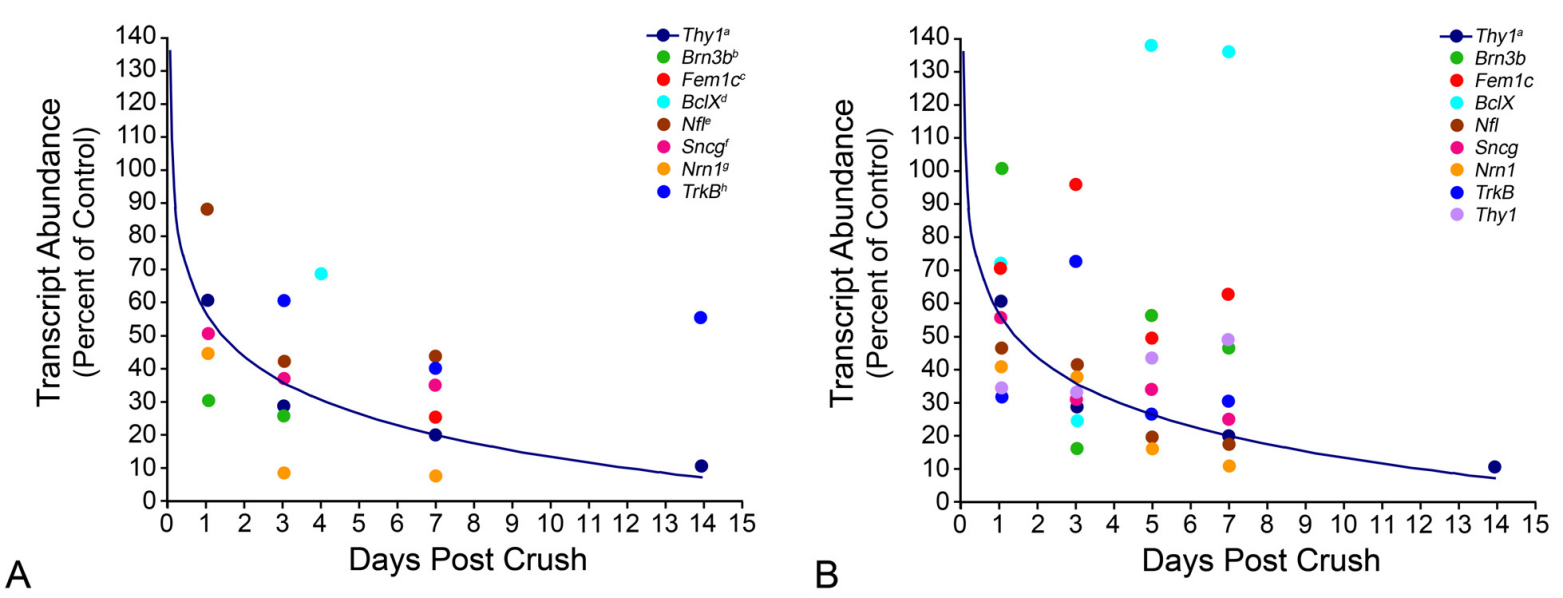
**Progressive gene silencing following optic nerve crush (ONC) precedes the loss of retinal ganglion cells (RGCs)**. Analysis of transcript levels of several silenced genes following ONC or optic nerve axotomy. (A) A retrospective analysis of published reports of gene silencing in apoptotic RGCs. Each data point represents the mean of values collected by different investigators to simplify the data set shown. One of the earliest demonstrations of gene silencing in RGCs after optic nerve damage was a quantitative RNase protection assay study for the RGC marker gene *Thy1 *[11]. These data points follow an exponential decay curve described by the equation, y = -18.441 Ln(x) + 55.703. All other data points are shown relative to this curve and displayed similar kinetics of silencing. Alternatively, during the first 7 days after ONC less than 1% of the cells had been eliminated (data not shown) [11]. Thus, gene silencing precedes cell loss by several days and represents a relatively early event in the apoptotic pathway. (B) Quantitative PCR analysis of silenced genes following ONC, graphed over the exponential decay curve of the original *Thy1 *studies. A similar decline in transcripts is evident in a more controlled prospective study. Interestingly, after an initial decline, *BclX *levels recover, consistent with earlier reports. In addition, genes with increases or no change in expression were also examined. Both *Gap43 *and *Bim *exhibited increases in transcript level in the experimental eye following ONC (145% and 116%, respectively), while *S16 *remained unchanged (99.8%, data not shown). In independent qPCR experiments, *cJun *transcript levels were also observed to increase modestly, shortly after ONC (112%). ^a ^[11] Transcript abundance quantified by RNase protection assays in mouse ONC. ^b ^[15] Quantified by qPCR in a rat model of axotomy. ^c^[12] Protein levels quantified by ELISA assays in mouse ONC. ^d^[14] Transcript abundance quantified by RNase protection assay in rat ONC. ^e, f, g^[5] Quantified by qPCR following axotomy in rats. ^h^[17] Transcript abundance measured by quantitative in situ hybridization studies following axotomy in rats.

**Figure 9:**
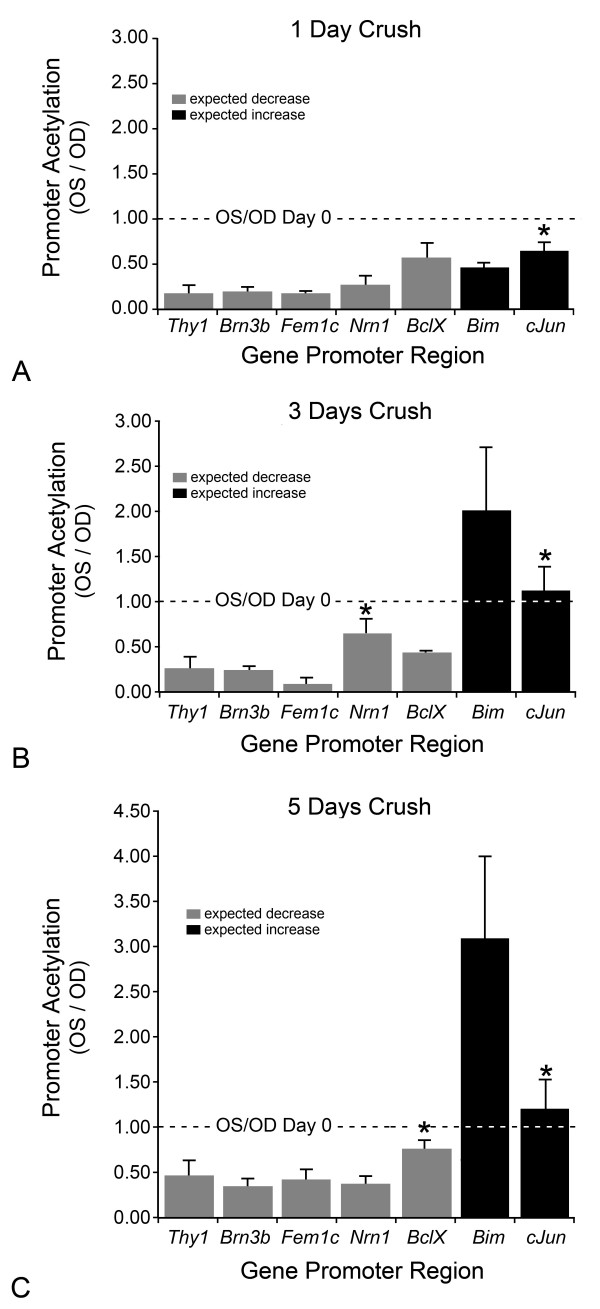
**Gene promoter associated histone H4 becomes deacetylated following optic nerve crush (ONC)**. Two gene sets were examined by chromatin immunoprecipitation for acetylated H4. The first was comprised of genes whose expression had been documented to decrease during ganglion cell apoptosis (grey bars), including *Thy1*, *Brn3b*, *Fem1c*, *Nrn1*, and *BclX *(see text). The second set consisted of a pair of genes (*Bim *and *cJun*) whose expression has been documented to increase during apoptosis (black bars). The data is shown as a ratio of crush (OS)/control (OD) and has been normalized to day 0 levels (dashed line) (n = 6 samples at each time point). (A) At 1 day post ONC, there was a significant decrease in promoter H4 acetylation of most of the genes examined (range of P values was 9.9 × 10^-5 ^for *Thy1 *to 0.04 for *Bim*). Promoter acetylation for *cJun *was decreased, but this was not significant compared to day 0 levels (*P = 0.08). (B) At 3 days post ONC, the decrease in promoter acetylation persisted for down-regulated genes. Conversely, the promoters of genes that are typically up-regulated exhibited a detectable increase in H4 acetylation to normal or above-normal levels. All changes were significantly different compared to day 0 levels, with the exceptions of *Nrn1 *and *cJun *(*P > 0.08). (C) The general pattern of H4 acetylation observed at 3 days post ONC, remained consistent through 5 days post ONC. Promoters for *BclX *and *cJun *exhibited no statistical change from day 0 histone acetylation levels at this stage (*P > 0.1).

To correlate the observed decrease in histone H4 acetylation with gene silencing, we determined if H4 deacetylation occurred in transcriptionally significant sites, such as the promoter regions of genes that are downregulated in RGCs in response to ONC. Following ONC, a number of genes are known to decrease in expression. A retrospective analysis of the literature indicated that the rapid decrease in transcript abundance for a majority of genes roughly follows an exponential decay curve (Figure 8A). To verify this in a controlled setting, we developed a mini qPCR array of several mRNAs expressed in RGCs and used it to monitor the change in transcript abundance in retinas over the first 7 days post ONC. Similar to the literature reports, this prospective study also showed the exponential decay of RGC transcripts (Figure 8B). Two sets of genes were examined for chromatin immunoprecipitation (ChIP) analysis. The first group included *Thy1*, *Brn3b*, *Nrn1*, and *Fem1c*, which are expressed predominately by RGCs in the retina, as well as the anti-apoptotic gene *BclX *[3-5,11,14,15]. This group of genes represented a subset of genes that exhibit a reported decrease in expression after ONC. The second group of genes, *Bim *and *cJun*, were examined because they undergo an increase in expression following ONC [5,18]. ChIP assays, with antibodies against acetylated H4, were performed on retinas from control and crush eyes followed by qPCR to quantify the genomic DNA that was collected. The quantification of promoter DNA associated with acetylated histone H4 is shown in Figure 9 and is expressed as a ratio of experimental: control retinas. All ratios have been normalized to day 0 controls. Surprisingly, promoter regions for both down-regulated and up-regulated genes showed a significant decrease in histone acetylation 1 day post ONC, with the exception of the promoter for *cJun *(Figure 9A). Three days post ONC, however, the acetylation pattern of promoter histones was changed and only down-regulated genes exhibited a decrease in promoter acetylation (Figure 9B), while promoter H4 acetylation for *cJun *had remained at day 0 levels and levels for *Bim *had significantly increased ~2-fold. A similar pattern of promoter acetylation observed 3 days after ONC was also evident on day 5 post ONC (Figure 9C), except that *Bim *H4 promoter acetylation had increased further to ~3-fold the level detected in day 0 retinas.

### Inhibition of HDAC activity blocks ONC-induced silencing of the *Fem1c^R3 ^*reporter gene

Although promoter histone deacetylation is associated with silenced genes in RGCs, these experiments do not conclusively demonstrate that this epigenetic change is the controlling mechanism for transcriptional downregulation. To address this, we examined if inhibitors of HDAC activity could block the ONC-mediated downregulation of RGC-specific gene expression. For these experiments, we used *Fem1c*^Rosa3 ^(R3) mice, which contain the βGeo promoter trap reporter in the first intron of the *Fem1c *gene. Previously, we showed that mice express βGEO in an RGC-specific manner [12]. Additionally, we have observed a 75% decrease in βGEO total protein and enzyme activity [12], and a 50% decrease in *Fem1c *transcript levels, by 5 days post ONC (Figure 8A, B). Thus, the R3 reporter allows for the rapid detection and quantification of changes in RGC gene expression.

**Figure 10:**
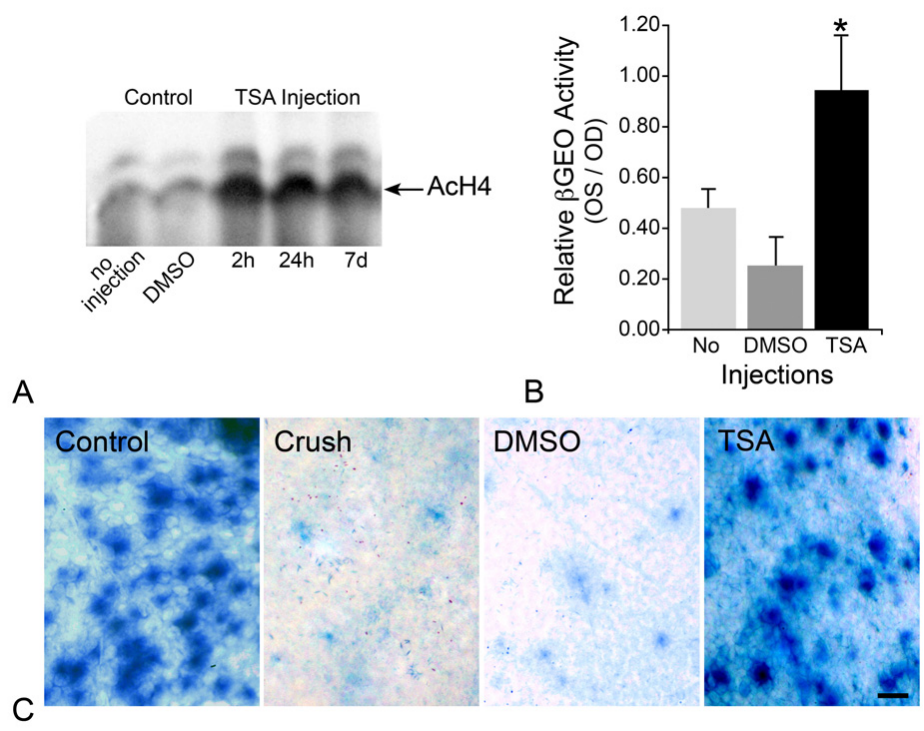
**Inhibition of histone deacetylase activity prevents the silencing of a reporter gene after optic nerve crush (ONC)**. (A) Western blot analysis of whole retinal acetylated histone H4 (AcH4) following intraperitoneal injection of trichostatin A (TSA) or vehicle control (DMSO). The times indicated are the time elapsed between injection of TSA and collection of the retinas. TSA resulted in hyperacetylation of H4 at all time points after treatment. (B) Retinal ganglion cell (RGC) specific gene expression was monitored as a function of βGalactosidase activity from the βGeo gene trap cassette inserted into *Fem1c *(see Methods). Histograph showing total retinal βGalactosidase activity 5 days post ONC. Mean ± SE is shown (n = 8-12 retinas per condition). Retinas with no injection or after DMSO vehicle injection (i.p.) exhibited a 50-75% decrease in βGEO activity after ONC. Conversely, TSA treated mice exhibited significantly higher βGEO activity following ONC than with crush alone or crush with DMSO injection (*P = 0.015 and P = 0.003, respectively). (C) Retinal wholemounts showing histochemical staining of βGEO enzyme activity, 5 days after ONC. The midperipheral region of the superior quadrant of each retina is shown. In this experiment, eyes were treated by intravitreal injections of DMSO vehicle or TSA at the time of surgery. Control retinas (control) typically showed prominent labeling of RGCs. Retinas from eyes that underwent crush alone (crush) or crush with DMSO treatment (DMSO) both showed a marked loss of staining of RGCs. βGEO activity remained robust in eyes that received TSA injections. Scale bar = 25 μm.

HDAC activity in the retina was inhibited by pretreatment of mice with TSA given as a single intraperitoneal (i.p.) injection, 24 hours before ONC. Western blot analysis of AcH4 levels in extracts of total retinal protein (Figure 10A) confirmed inhibition of HDAC activity, which was exemplified by hyperacetylation of H4. The effects of TSA were detected as quickly as 2 hours after injection and persisted as long as 7 days post injection. R3 gene expression was assessed by βGEO solution assays 5 days post ONC (Figure 10B). ONC resulted in a 55-75% decrease in βGEO enzyme activity by 5 days after surgery. TSA treated mice, however, exhibited significantly more activity at this time point than mice receiving no injection or DMSO injections (P = 0.016 and P = 0.003, respectively). In a separate series of experiments, TSA was also administered as a single intravitreal injection at the time of ONC surgery. Whole-mounted retinas from these experiments exhibited βGEO staining in RGCs consistent with the solution assays (Figure 10C). Taken together, these data suggested that TSA was able to attenuate the silencing of the *Fem1c *gene.

### Inhibition of HDAC activity attenuates cell loss following ONC

**Figure 11:**
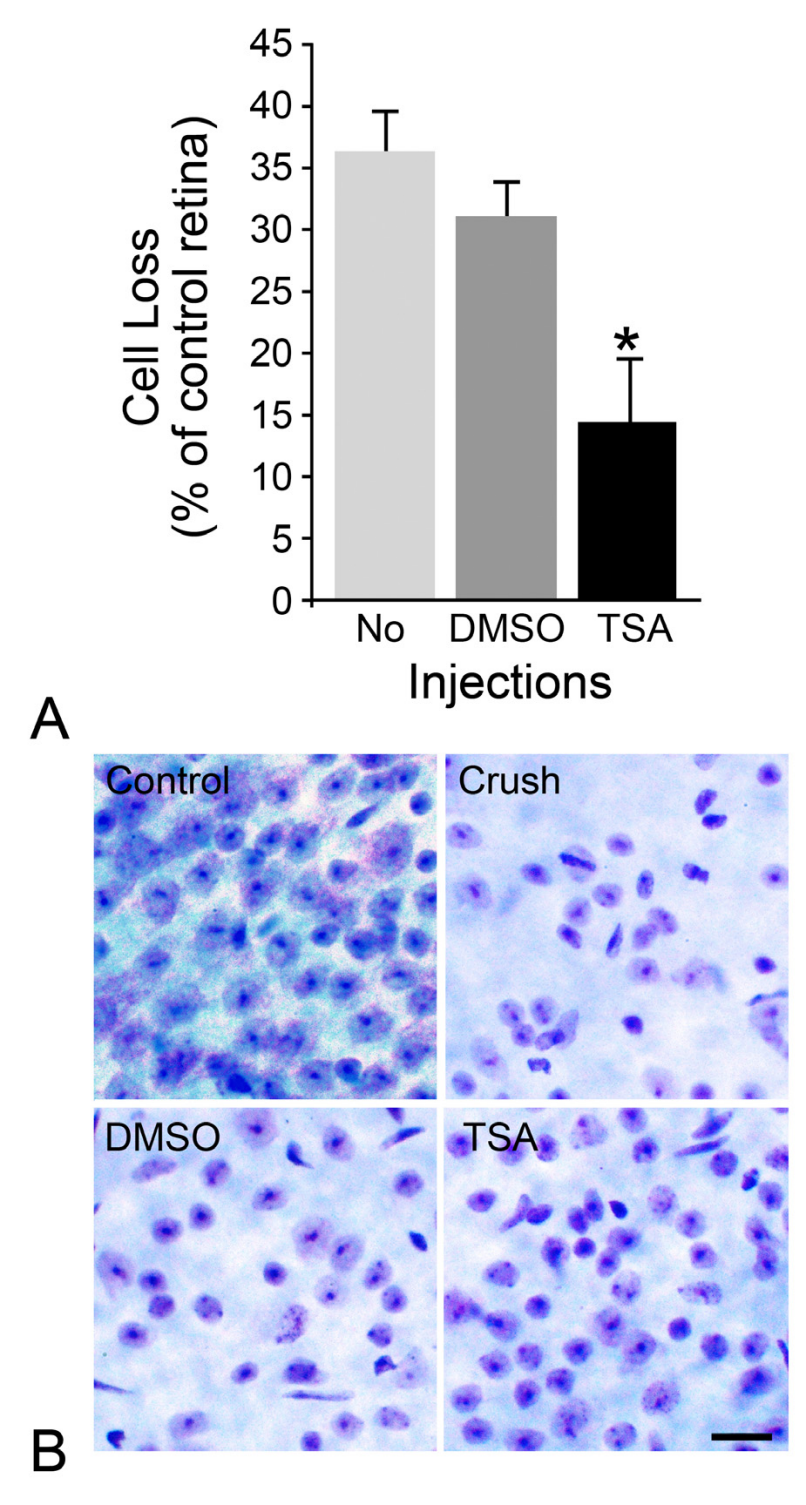
**Inhibition of HDAC activity attenuates cell loss following optic nerve crush (ONC)**. Total cell counts in the ganglion cell layer (GCL) were performed to determine the protective effect of HDAC inhibition with trichostatin A (TSA) following ONC. (A) Histograph showing cell loss in the GCL in crush eyes as a percentage of cells in the contralateral control eyes at 2 weeks post ONC. Mean ± SE is shown (n = 6-10 retinas per treatment condition). Mice that received no injection or an injection of DMSO exhibited a 36.4 and 31.2% cell loss, respectively. Mice that were given a TSA injection prior to ONC only exhibited a 14.4% cell loss, which was significantly less than that seen with no injection or DMSO (*P = 0.005 vs. crush alone, P = 0.01 vs. DMSO). (B) Representative Nissl stained whole mounts of the mid-peripheral region of mouse retinas 2 weeks after ONC. Retinas after ONC, crush alone (crush) or crush with DMSO (DMSO), exhibited a loss of cells compared to fellow control eyes (control). TSA treated mice (TSA) exhibited less cell loss after ONC, although cells in these retinas appeared smaller due to atrophy associated with optic nerve lesion, as described by others [53]. Scale bar = 20 μm.

Although histone deacetylation plays a role in modulating gene silencing during apoptosis, it is unknown if this process is a critical stage in the progression of the cell death program. To address this, we injected mice (i.p.) with the HDAC inhibitor, TSA, 24 hours prior to ONC. Retinas were then examined 2 weeks after surgery, which represents a point when there is normally significant cell loss [33]. The mice that underwent crush alone, or mice that received an injection of DMSO prior to ONC, exhibited comparable losses of 36.4 ± 3.4% and 31.2 ± 2.9% of cells in the GCL (P = 0.13, Figure 11A). Conversely, mice that received TSA prior to ONC showed a significant attenuation of cell loss in the GCL (14.4 ± 5.3%) as compared to both crush alone and crush with DMSO (P = 0.005 and P = 0.01, respectively). Representative Nissl-stained whole mounts of retinas from a control eye and each of the three treatments are shown in Figure 11B. Although cell loss was attenuated by treatment with TSA, surviving cells did exhibit signs of atrophy such as somal shrinkage.

## Discussion

Previous studies by our group and others have shown that silencing of normal gene expression is an early event in the apoptotic pathway of neurons, including RGCs [5,11,12,15]. Although microarray studies have carefully documented early gene expression changes in dying neurons, little attention has been given to understanding the causative mechanism leading to these widespread changes. Here we propose that epigenetic changes in active chromatin, specifically histone deacetylation, are part of the underlying mechanisms of apoptotic gene silencing.

We were able to detect an increase in whole retinal nuclear HDAC activity at the earliest time point examined (1 day post ONC), however, it was not significantly higher until day 5. This lag in HDAC activity may reflect that the increase was mainly occurring in the RGCs, which only comprise 1-2% of the cell population in the retina. Therefore, day 5 post ONC may represent a point when a maximum number of RGCs were exhibiting an increase in HDAC activity. Conversely, because this experiment was performed on whole retina extracts and not on RGC-enriched samples, the increase in activity could possibly be due to changes in other cell types within the retina. The immunofluorescent studies examining changes in nuclear histone H4 acetylation, however, suggest that the changes in HDAC activity are likely limited to dying cells in the GCL.

**Figure 12:**
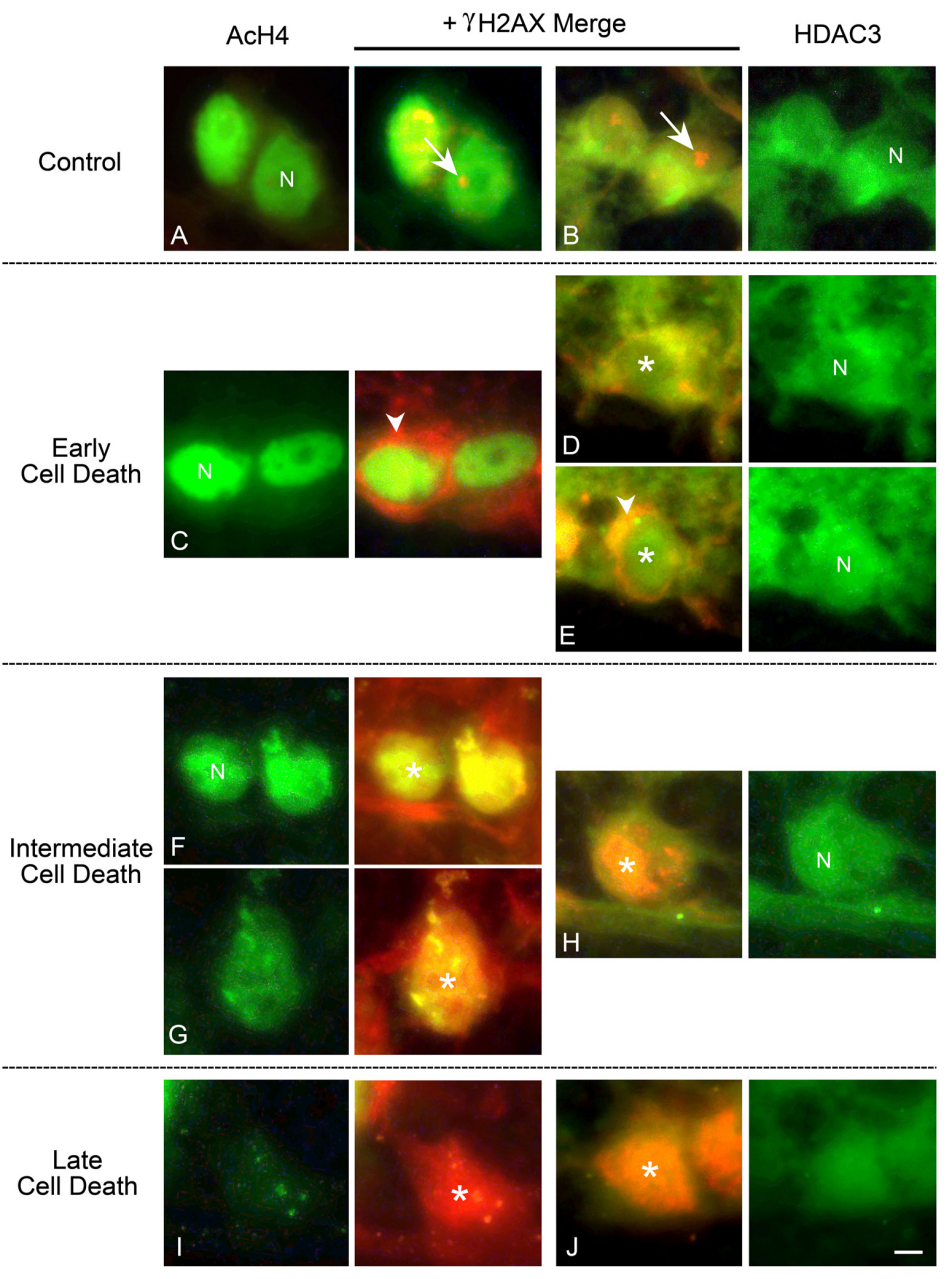
**HDAC3 becomes localized to nuclei prior to the deacetylation of histone H4 in dying cells**. Retinal cryosections before and after optic nerve crush (ONC) were colabeled with either γH2AX and AcH4 or HDAC3 antibodies. Representative cells from the ganglion cell layer (GCL) are shown for four stages of γH2AX localization. (A, B) Control retinas showed nuclear labeling of acetylated H4 (AcH4) and cytoplasmic labeling of HDAC3. Representative nuclei are indicated (N). γH2AX was present as a densely labeled spot associating with the nucleoli (stage I labeling, Figure 5). (C) Early in the cell death process, nuclei (N) in the GCL were still brightly labeled for AcH4. At this stage, γH2AX had begun to appear as a perinuclear ring (arrowhead) (stage II labeling) and was not colocalized with the nuclear AcH4. Conversely, γH2AX did colocalize with HDAC3 at this stage. (D, E) These images illustrate the progressive movement of HDAC3 into the nuclei of dying cells. (D) HDAC3 was mainly present in the cytoplasm, but appeared to be concentrating around the nucleus (N). In E, HDAC3 was distributed in both the nucleus (asterisk in merged image), and colocalized with perinuclear γH2AX (arrowhead). (F, G) After HDAC3 had begun to accumulate in the nuclei, a progressive decrease in AcH4 labeling was detected. Also at this point, γH2AX was present in the affected nuclei (stage III labeling, asterisk in G). (H) HDAC3 labeling continued to increase in the nuclei and was now colocalized with nuclear γH2AX (asterisk). (I, J) At this late stage of cell death, there was virtually no AcH4 left in the γH2AX-labeled cells (asterisk in I), while γH2AX and HDAC3 remained colocalized in the nuclei (asterisk in J). A graphic representation of these changes is also shown in additional file 2. Scale bar = 2 μm.

Our experiments suggest that HDACs 2 and 3 play a central role in the process of histone deacetylation during RGC death. We have focused on HDAC3, principally because there is a sustained increase in transcripts for this gene after ONC, and HDAC3 protein translocates to nuclei of dying cells. It is reasonable to speculate that the movement of HDAC3 is a principal mechanism that increases nuclear HDAC activity in RGCs, and that this leads to the deacetylation of histone H4. We can infer the sequence of events defining the relationship between HDAC3 and the deacetylation of H4 by using the expression and localization of γH2AX to identify the stage of the apoptotic pathway that any given cell is in (Figure 12 and additional file 3: The change in expression and cellular distribution of γH2AX provides a temporal indicator of histone deacetylation in damaged ganglion cells). Early in dying cells (stage II γH2AX), cytoplasmic HDAC3 appears to translocate to the nucleus in advance of γH2AX nuclear localization. This is evident by cells, which exhibit stage II labeling but may be either cytoplasmic or nuclear for HDAC3. The majority of these cells are present by day 1 post ONC, and already show a quantifiable decrease in AcH4 (Figure 4). Additionally, qPCR data show significant increases in *Hdac *2 and 3 transcripts by this day, and ChIP analysis shows significant deacetylation of target promoters coincident with a dramatic decrease in transcript abundance from these target genes (Figures 8 and 9). Thus, by all accounts, a localized increase in nuclear HDAC activity that leads to gene silencing by promoter histone deacetylation appears to be a very early event in the response of the RGC soma after ONC.

The question that remains, however, is why does there appear to be a progressive increase in the level of nuclear HDAC activity and a similar progressive decrease in histone deacetylation after this initial silencing event? Part of the answer to this may lie in the relative sensitivity of the different assays used to detect changes in transcript abundance, promoter acetylation, HDAC activity, and H4 acetylation levels, but more likely the consequences of deacetylation are two-fold. The early onset of deacetylation may selectively target actively expressed genes, leading to gene silencing. Later, progressive and global deacetylation may be required as the cell continues through the apoptotic pathway, in order to facilitate the condensation of the nuclear chromatin into a heterochromatic state. Chromatin condensation is one of the morphological hallmarks of apoptosis, and has been clearly documented in apoptotic RGCs [34,35]. Histone tails in condensed heterochromatin are generally hypermethylated and hypoacetylated [22]. HDAC3, in particular, may play a key role in chromatin condensation. Previous studies have shown that deacetylation of histone H3 by HDAC3 initiates chromatin condensation during mitosis by creating a preferred binding site for Aurora B kinase, which then phosphorylates H3 [36]. This modification of the histone code is the first of several events that eventually lead to chromatin condensation.

Although our experiments show that deacetylation of histones may be a central mechanism of transcriptional silencing, consistent with other reports [37,38], they do not exclude involvement of other chromatin modifications such as the methylation and demethylation of histones. Unlike the association between acetylation status of histones and transcriptional activity, the role of methylation in regulating transcription is more complicated. Trimethylation of lysine 4 in histone H3 (H3K4me3), for example, is associated with transcriptional activity, while trimethylation of H4K20 is associated with silent chromatin [24,39]. Additionally, HDAC activity is often closely linked to the activity of demethylases, since these enzymes are part of larger protein complexes. In both humans and mice, HDAC2 is often part of the REST complex, which also includes the histone demethylases RBP2 and AOF2 [40]. Similarly, HDAC3 is a component of the SMRT/N-CoR complex, which is able to associate with the histone demethylase JMJD2A [41]. Preliminary RT-PCR results from our laboratory have shown that transcripts for both *Ncor1 *and *Ncor2 *(the mouse homologs to *SMRT and N-COR*), as well as *Rcor2 *(*COREST*) and *Aof2*, are present in the mouse retina (data not shown), indicating that the components for active HDAC3 and HDAC2 complexes are expressed in this tissue.

### Modulation of the HAT and HDAC activity balance during neurodegeneration

In healthy cells, HAT and HDAC activities are balanced to regulate transcriptional activity [24]. The disruption of this balance, as demonstrated through the use of HDAC inhibitors, can lead to apoptosis principally in rapidly dividing cells [42,43]. Disruption of the HAT:HDAC balance also appears to play a role in neurodegenerative diseases, albeit by a mechanism that appears to be different from the lethal imbalances that cause cancer cell death. Rather than decreases, relative increases in HDAC activity contribute to the progression of neuronal apoptosis in several disease models [37,44,45]. One of the consequences of this imbalance could be an overall decrease in histone acetylation, which leads to a decrease in gene expression. Studies in which HDAC activity is suppressed by HDAC inhibitors, presumably restoring the HAT:HDAC balance, show that this treatment is able to attenuate neuronal apoptosis. Several groups investigating models of polyglutamine expansion neurodegenerative diseases, such as Huntington's disease, for example, have used HDAC inhibitors to prevent cell loss [38,46-48]. In these models, the relative increase in HDAC activity has been hypothetically attributed to a decrease in HAT activity resulting from the sequestration and degradation of the acetyltransferase, CREB binding protein (CBP) [38,46], while HDAC activity levels remain unchanged [49]. This model of neurodegeneration implies that the relative increase in HDAC activity is a passive consequence of the selective loss of CBP. Our data, although consistent with the idea that HDAC activity is relatively increased, suggest that this change is reflective of an active increase in nuclear HDAC levels in dying RGCs, associated with both a modest increase in HDAC gene expression and the translocation of an active protein. In fact, nuclear HAT activity assays show no significant changes in overall acetyltransferase activity in retinas harvested from eyes after ONC (data not shown).

The overall importance of HDAC activity in the process of RGC death can be probed with HDAC inhibitors such as TSA. TSA pretreatment was able to attenuate the down regulation of at least one gene normally expressed in RGCs (*Fem1c^R3^*). Consistent with the other reports showing a protective effect in models of neurodegeneration, TSA was also able to provide a modest protective effect to RGCs after ONC. The underlying cause for this latter effect is not known and may be due to a variety of factors, such as stabilizing the balance between HAT and HDAC activity or allowing for increased acetylation of factors such as Sp1, which has been shown to be neuroprotective in a model of hypoxic stress [50]. Conversely, it is equally possible that maintaining normal gene expression may have a secondary protective effect to the RGCs. For example, previously we showed that the anti-apoptotic gene *BclX *was down-regulated after optic nerve crush in rats [14]. Preventing this decrease could result in an increase in RGC resistance to a damaging stimulus by antagonizing the actions of proteins like BAX.

Although several studies have linked an increase in HDAC activity to neuronal death, others have demonstrated that the overexpression of HDACs can be neuroprotective. Chen and Cepko showed that the overexpression of HDAC4 led to increased protein stability of the transcriptional activator HIF1α, which had a protective effect in a model of photoreceptor degeneration [51]. A related study demonstrated that part of the beneficial effect of HDAC4 on HIF1α transcriptional activity was due to HIF1α's increased ability to bind the histone acetyltransferase, p300 [52], which could be beneficial during neurodegeneration when fewer active HATs are available [37,44]. In these cases, the protective effect of increased HDAC activity appeared to be restricted to cytoplasmic activity on non-histone substrates.

Irrespective of the principal function of HDAC activity during cell death, the phenomenon of gene silencing is likely going to act as a barrier to regaining normal cell function in neuroprotective strategies. *Bax*-deficient RGCs, which are completely resistant to apoptosis after ONC, for example, can remain in a genetically silent, heterochromatic state for months following injury (unpublished data). Similarly, inhibition of HDAC activity using TSA, still resulted in some cell atrophy characterized by soma shrinkage. Shrinkage of RGCs after optic nerve damage has been described by others [53], including in *Bax*^-/- ^RGCs [54]. This atrophy response may be indicative of the apoptotic volume decrease described in some neuronal cell types [55], which is considered to be a very early event in the apoptotic program and is regulated by a rapid efflux of intracellular potassium in many neurons, including retinal ganglion cells [56,57]. The effects we observe in TSA-treated mice suggest that epigenetic changes leading to gene silencing is downstream of the apoptotic volume decrease. Nevertheless, a complete understanding of the early changes in affected ganglion cells remains an important consideration for neuroprotective strategies. Clearly, regaining normal cell function will ultimately require reactivation of normal gene expression, meaning reversal of epigenetic changes associated with silenced genes.

## Conclusions

In summary, this study demonstrates that a change in histone acetylation levels, particularly in the promoter regions of silenced genes, may be part of the underlying mechanism of apoptotic gene silencing. In addition, there is some evidence indicating that HDAC3 may be the predominant HDAC isoform responsible for apoptotic histone deacetylation in dying RGCs. Initial HDAC inhibition studies indicate that TSA can prevent gene silencing and has a neuroprotective effect, suggesting that histone deacetylation may be a critical stage in the apoptotic pathway.

## Methods

### Experimental animals and optic nerve crush

All mice were handled in accordance with the Association for Research in Vision and Ophthalmology statement for the use of animals for research. The majority of experiments were conducted on CB6F1 mice, which have been used in the past by our group to quantify cell loss and changes in RGC transcript levels, as a result of ONC. In some experiments, however, ROSA3, a substrain of C57BL/6 mice [12], were used to utilize the expression of βGEO marker protein as a way to more precisely quantify RGC-specific gene expression. These latter mice express βGEO as the result of transcription of the *Fem1c *gene, which is predominantly RGC-specific in the retina. They also exhibit similar kinetics of cell loss observed in the CB6F1 strain. For both strains, a random mixture of males and females between the ages of 4-6 months were used. ONC was performed unilaterally as described previously to initiate degeneration of the retinal ganglion cells [30]. Retinas were harvested 1, 3, 5, 7, or 14 days post ONC as indicated.

In some cases, retinal ganglion cells were retrogradely labeled with the tracer dye, fluorogold (Molecular Probes, Eugene, OR). Labeling was performed by first exposing the superior collicli on each side of the brain and placing a small piece of gel-foam, soaked in 0.9% NaCl containing 2% fluorogold, to each exposed surface. ONC surgery was performed 3 days after dye application.

### HDAC activity assay and nuclear protein extraction

Nuclear proteins were extracted from whole retinas according to Andrews and Faller (1991). Protein concentration was determined using a BCA protein assay kit (Pierce, Rockford, IL). HDAC activity assays were performed using a Fluor de Lys kit (BIOMOL International, Plymouth Meeting, PA). Triplicate samples containing 4 μg of protein each were loaded in an opaque 96-well plate with Fluor de Lys substrate at a final concentration of 150 μM. Following a 20 minute incubation at room temperature, 1 × Fluor de Lys developer with trichostatin A was added to stop the reaction and develop the fluorescent signal. Plates were read using a CytoFluor plate reader at 360 nm excitation and 440 nm emission wavelengths (Perkin Elmer, Waltham, MA). All samples were corrected to a buffer-only blank and normalized to the HeLa extract controls.

### Western blot analysis

Western blot analysis on nuclear and cytoplasmic retinal protein fractions (see above) was performed as previously described [58] with the following modifications. Each fraction was loaded as a single lane (500 μg) on separate12% polyacrylamide gels and transblotted onto Immobilon P (Millipore, Inc, Billerica, MA). Membranes were stained with Ponceau S, cut into strips, and probed with various antibodies. Rabbit polyclonal antibodies for HDACs 1-5 (Santa Cruz, city CA) and anti-acetyl-histone H4 ChIP grade rabbit antiserum (Millipore, Inc) were used at a 1:100 dilution. Goat anti-rabbit secondary antibodies, conjugated to alkaline phosphatase, were used at a 1:500 dilution (Jackson ImmunoResearch Laboratories Inc, West Grove, PA). Blots were color developed using NBT and BCIP and digitally scanned.

### Evaluation of transcripts in the retina by quantitative PCR

Total retinal RNA was isolated at 1, 3, and 7 days post ONC and 2 μg was used for cDNA synthesis with reverse transcriptase and oligo(dT) [11]. The resulting cDNA was diluted 10-fold and 1 μl was used for each qPCR reaction with SYBR Green PCR master mix (Applied Biosystems, Foster City, CA) and the appropriate HDAC primers (Table 1), as described previously [59]. Each primer set was optimized, and the resulting amplimer cloned and sequenced to confirm identity. Quantitative PCR was conducted on triplicate samples in each run using an ABI 7300 Real Time PCR system (Applied Biosystems). Transcript quantification was based on standard curves of each target amplimer and the absolute value for copy number was normalized to *S16 *ribosomal protein mRNA. The values are expressed as the ratio of crush: control and normalized to the day 0 ratio that was set at 1.

**Table 1:** Primers for ChIP and qPCR analysis

Gene Name	Primer Sequences	Size of product (bp)	**Position**^**a**^
*Hdac1*	5'-GTCAAAGGAGCCCACGCCAG-3'	603	+555/+1157
	5'-GTTGACAGCTTCGGGGAGGC-3'		
*Hdac2*	5'-ATGGCGTACAGTCAAGGAGG-3'	359	+209/+568
	5'-AGCAACTGAACCACCCGTGG-3'		
*Hdac3*	5'-GTATGACAGGACTGACGAGG-3'	168	+528/+695
	5'-TTTCCTTCCCACCACAGAGG-3'		
*Hdac4*	5'-CATCCCCAAGCCAAGCGAGC-3'	619	+1521/+2140
	5'-CCGTCTGTAGCTCCTCCAGG-3'		
*Hdac5*	5'-CTGTGCCTCATCAGGCCCTG-3'	642	+1919/+2560
	5'-CCACGCTCAGCTTCTGCTGC-3'		
*Thy1 *	5'-CTGGAATCAAAGGTGTGAGC-3'	190	-288/-98
promoter	5'-CCCCTCTCTTTATCCCCTTC-3'		
*Brn3b *	5'-CTGAGGTCTGAAGCCAGAGC-3'	266	+17/+282
promoter	5'-ACGCCTGCTTGCTGTTCAGG-3'		
*Fem1c *	5'-TCCGCAGCCTTTCAGGATGC-3'	188	-255/-67
promoter	5'-AGTGCGCCTGCGTACTAAGG-3'		
*Nrn1 *	5'-AACAATCGAGTGGGCGCACC-3'	251	-137/+114
promoter	5'-AAGTACAGCCTCACGCCCACG-3'		
*BclX *	5'-ATCTGGTCGATGGAGGAACC-3'	214	+93/+326
promoter	5'-AATCTATCTCCGGCGACAGC-3'		
*Bim *	5'-AAAGCAAGGGCGGAGGGACG-3'	238	-369/-132
promoter	5'-CTGTCCTGCAGTTTGCGTGC-3'		
*cJun *	5'-AACACAAGCCGAAGCTGAGC-3'	263	-265/-2
promoter	5'-AGTCCTTATCCAGCCTGAGC-3'		

^a ^Indicates position (in base pairs) relative to the transcriptional start site (+1)

In addition to evaluating individual transcripts in separate experiments, a qPCR mini array was developed that allowed us to examine 15 different mRNAs simultaneously during a single run. Each 96-well plate of the array contained triplicate assays for 5 RGC-specific genes (*Thy1, Brn3b, Scng, Nrn1, Fem1c*), 4 genes that are abundantly expressed in RGCs, and reportedly decline in expression, but are not necessarily RGC-specific (*TrkB, Nfl, BclX, Tubb3*), 4 genes whose expression likely increase in damaged retinas (*Bim, Gap43, Hsp27, Gfap*), and 2 control genes (*Gad67 *and *S16*). Two complete samples could be simultaneously examined on each plate. Primer design (Table 2) and PCR conditions were first normalized so that efficient amplification was obtained for all targets under the same PCR conditions. Sample quantification was carried out as described above, using a common standard curve for the entire plate.

**Table 2:** Primers for qPCR mini array analysis

Gene Name	Primer Sequences	Size of product (bp)
*Thy1*	5'-CTTGCAGGTGTCCCGAGGGC-3'	379
	5'-CTGAACCAGCAGGCTTATGC-3'	
*Brn3b*	5'-TCTTCCAACCCCACCGAGC-3'	157
	5'-GTGGTAAGTGGCGTCCGGCTTG-3'	
*Sncg*	5'-GACCAAGCAGGGAGTAACGG-3'	240
	5'-TCCAAGTCCTCCTTGCGCAC-3'	
*Nrn1*	5'-TTCACTGATCCTCGCGGTGC-3'	238
	5'-TACTTTCGCCCCTTCCTGGC-3'	
*Fem1c*	5'-GAAGTGTCCAACCGCCATGG-3'	292
	5'-TTGTCTGGGCATGGTGCG-3'	
*TrkB*	5'-GTCTGACCTGATCCTGACGG-3'	280
	X5'-CCCAACGTCCCAGTACAAGG-3'	
*Nfl*	5'-AGCACGAAGAGCGAGATGGC-3'	173
	5'-TGCGAGCTCTGAGAGTAGCC-3'	
BclX	5'-TTGGACAATGGACTGGTTGA-3'	780
	5'-GTAGAGTGGATGGTCAGTG-3'	
*Tubb3*	5'-GTTCTGGGAGGTCATCAGCG-3'	207
	5'-TCGGGCCTGAATAGGTGTCC-3'	
*Bim*	5'-TCTGAGTGTGACAGAGAAGG-3'	378
	5'-CTCCTGAGACTGTCGTATGG-3'	
*Gap43*	5'-TGAGCAAGCGAGCAGAAA-3'	199
	5'-GCAGCCTTATGAGCCTTA-3'	
*Hsp27*	5'-CGCAACAGCAGTCATGTCGG-3'	257
	5'-GGCTCACATCCAGAAACGCC-3'	
*Gfap*	5'-CAAACTGGCTGATGTCTACC-3'	269
	5'-AGAACTGGATCTCCTCCTCC-3'	
*Gad67*	5'-TCTTCCACTCCTTCGCCTGC-3'	279
	5'-GGAGAAGTCGGTCTCTGTGC-3'	
*S16*	5'-CACTGCAAACGGGGAAATGG-3'	198
	5'-TGAGATGGACTGTCGGATGG-3'	

All primer pairs were designed to span at least one intron.

### Immunofluorescence and quantification of AcH4

Indirect immunofluorescence on 5 μm thick retinal cryosections was done as described previously [60]. Primary antibodies included γH2AX (monoclonal, Millipore, Inc), HDAC2, HDAC3, and AcH4 (previously mentioned) and were used at a 1:100 dilution. Secondary antibodies used were goat anti-rabbit with a Texas Red label (1:100) and goat anti-mouse with a FITC label (1:100) (both from Jackson ImmunoResearch Laboratories). The images were obtained using a Zeiss Axioplan 2 Imaging microscope with Axiovision 4.6.3.0 software (Carl Zeiss MicroImaging, Inc., Thornwood, NY) and viewed in Adobe Photoshop.

To quantify AcH4 staining, nuclear pixel intensity was measured in the ganglion cell (GCL) and inner nuclear layers (INL) using the outline function of the Zeiss Axiovision software. A minimum of 99 cells was counted in each layer and the mean pixel intensity per nucleus was calculated as a function of the nuclear area (in μm^2^). The calculated pixel intensity of GCL nuclei was normalized to the calculated pixel intensity of nuclei of the INL and the final calculated intensity was expressed as a ratio of crush:control.

### Chromatin immunoprecipitation (ChIP) assays

Acetyl-histone H4 ChIP assays were performed as outlined by the manufacturer (Millipore). In each assay, 6 retinas were pooled and half of each sample was mixed with either AcH4 antibody or normal rabbit serum for a control (Jackson ImmunoResearch Laboratories). The supernatant obtained from the normal serum samples following immunoprecipitation was regarded as the input. DNA from immunopreciptates was unlinked from protein complexes and purified further by phenol/chloroform extraction. Samples were analyzed in triplicate using qPCR as described above. The data obtained from qPCR were analyzed by subtracting the normal serum samples from the AcH4 immunoprecipitated samples and converting this to a percentage of the total input. These numbers were then expressed as a ratio of crush:control and normalized to the day 0 values.

### HDAC inhibitor studies

To inhibit HDAC activity in the retina, TSA was injected either intraperitoneally (1 mg/kg in DMSO) 24 hours prior to ONC surgery or intravitreally (1 μl of 20 μM TSA in DMSO) immediately after ONC surgery. Vehicle injections consisted of an equal volume of DMSO. The effects of TSA on gene expression were conducted on *Fem1c^R3/+ ^*mice, which express the βGEO enzyme predominantly in RGCs in the retina. The level of βGeo expression was analyzed using two methods. Firstly, the level of βGEO activity in individual retinas was quantified by β-Galactosidase solution assay (Promega, Madison, WI) 5 days post ONC. The plates were read with an EL_X_800 microplate reader (Bio-Tek Instruments Inc., Winooski, VT). Duplicate samples of each eye was measured and total activity was calculated after subtraction of the β-Galactosidase activity measured in wild-type mice and corrected to the amount of total protein loaded in each sample. Secondly, βGEO expressing cells were identified histochemically, 5 days post ONC, by staining retinal preparations with X-Gal, followed by whole mounting as previously described [12]. To assess the effects of TSA on RGC loss, a 1 mg/kg intraperitoneal injection of TSA was administered 24 hours prior to ONC of adult CB6F1 mice. Two weeks after ONC, cells in the GCL were Nissl-stained and counted and compared to controls [33].

### Statistical analysis

Data was collected from a minimum of 3 independent samples in all experiments, and shown as the mean ± Standard Error in graphs. All statistical analyses were performed using the Student's *t*-test with statistical significance set at P ≤ 0.05.

## Authors' contributions

HRP participated in the design of this experiment, conducted the HDAC activity assays, immunofluorescence studies, ChIP assays, qPCR experiments, wholemount staining, cell counts, analyzed the data, and drafted the manuscript. CLS performed the Western Blot analysis, assisted with the immunofluorescence experiments, analyzed the data, and contributed to the drafting of the manuscript. RWN conceived of this experiment, performed the optic nerve crush, and helped draft the manuscript. All authors read and approved the final manuscript.

## Supplementary Material

Additional file 1 **Localization of HDACs 2 and 3 before and after optic nerve crush**. Photomicrographs of the individual channels for the merged images of Figure 3B. Sections from control retinas and retinas 3 days after optic nerve crush (ONC) were labeled with antibodies against HDAC2 or HDAC3 and counter-stained with DAPI to highlight nuclei. High-magnification images of the GCL are shown. (A, B) In control and crush retinas, HDAC2 labeling (red) was present in the nuclei of cells in the GCL, as determined by DAPI staining (blue). (C) In control retinas, HDAC3 labeling had a diffuse, cytoplasmic appearance with minimal overlap with nuclear DAPI staining. (D) At 3 days post ONC, many cells in the GCL exhibited nuclear HDAC3 labeling that coincided with DAPI staining of the nuclei. Scale bar = 5 μm. Click here for file

Additional file 2 **The deacetylation of histone H4 occurs in cells with nuclear γH2AX and DNA fragmentation**. Sections from a retina after optic nerve injury were double labeled with antibodies against acetylated histone H4 (AcH4, green) and γH2AX (red), to identify apoptotic cells, and counter-stained with DAPI (blue) to verify the nuclear presence of the proteins. Four cells in the ganglion cell layer are visible in various stages of cell death. DAPI-staining of 3 of the cells show condensed and fragmenting nuclei, consistent with apoptosis (arrows). One cell exhibits a normal nuclear staining pattern, including the presence of a robust nucleolus. Two of the cells with condensed chromatin also exhibit stage III γH2AX staining and all 3 cells are weakly staining for AcH4. The relatively normal appearing cell exhibits both strong stage II γH2AX (arrowhead) and AcH4 staining. A single representative nucleus, showing of DNA fragmentation and stage III γH2AX labeling is indicated (N). Size bar = 5 μm. Click here for file

Additional file 3 **The change in expression and cellular distribution of γH2AX provides a temporal indicator of histone deacetylation in damaged ganglion cells**. A graphic representation of the co-localization of γH2AX staining and HDAC3 or Acetyl H4 (AcH4), indicates that HDAC3 is translocated into nuclei of apoptotic cells before the loss of AcH4 staining. The top row of cells represents the co-localization of γH2AX with HDAC3, while the bottom row represents the co-localization of γH2AX with AcH4. The labeling pattern of just γH2AX is shown in the center row of cells. In each row, γH2AX staining is depicted in red, while HDAC3 and AcH4 are depicted in green. At day 0, cells are negative for γH2AX staining, with the exception of a spot adjacent to the nucleolus. These cells are classified as stage I, and exhibit cytosolic staining for HDAC3 and nuclear staining for AcH4. Once apoptosis is initiated, γH2AX-staining appears as a perinuclear ring (stage II, principally detected at day 1 after optic nerve crush), which appears yellow in some cells co-stained for HDAC3 (Figures 6B and 12C), indicating complete co-localization. Some cells, however, appear to have a more orange ring with a green nucleus, suggesting movement of HDAC3 from the cytosol to the nucleus in stage II cells (Figures 6B asterisk, 6C, 12E). In cells with nuclear γH2AX-staining (stage III, detected in increasing amounts at 3, 5, and 7 days post optic nerve crush), cells co-labeled for HDAC3 exhibit yellow to orange nuclei, indicating co-localization of these proteins (Figures 6D-F, 12H, 12J). With respect to AcH4 labeling, stage I cells exhibit bright green nuclei. Stage II cells typically appear as red rings of γH2AX label surrounding green nuclei (Figures 7B, 12C). Stage III cells exhibit three different nuclear colors, indicative of the extent of AcH4 label present, and appear to progress from yellow (Figures 7D, 12F), to orange (Figures 7E, 12G), to red (Figures 7F, 12I). Orange to red nuclei are often misshapen, although DAPI staining confirms the presence of condensed and fragmented DNA in these structures (See additional file 2). Click here for file

## References

[B1] Chang LK Putcha GV Deshmukh M Johnson EM Jr Mitochondrial involvement in the point of no return in neuronal apoptosis Biochimie 2002 84 2-3 223 231 10.1016/S0300-9084(02)01372-X 12022953

[B2] Cha JH Transcriptional dysregulation in Huntington's disease Trends Neurosci 2000 23 9 387 392 10.1016/S0166-2236(00)01609-X 10941183

[B3] Ahmed F Brown KM Stephan DA Morrison JC Johnson EC Tomarev SI Microarray analysis of changes in mRNA levels in the rat retina after experimental elevation of intraocular pressure Invest Ophthalmol Vis Sci 2004 45 4 1247 1258 10.1167/iovs.03-1123 15037594

[B4] Soto I Oglesby E Buckingham BP Son JL Roberson ED Steele MR Inman DM Vetter ML Horner PJ Marsh-Armstrong N Retinal ganglion cells downregulate gene expression and lose their axons within the optic nerve head in a mouse glaucoma model J Neurosci 2008 28 2 548 561 10.1523/JNEUROSCI.3714-07.2008 18184797 PMC6670511

[B5] Yang Z Quigley HA Pease ME Yang Y Qian J Valenta D Zack DJ Changes in gene expression in experimental glaucoma and optic nerve transection: the equilibrium between protective and detrimental mechanisms Invest Ophthalmol Vis Sci 2007 48 12 5539 5548 10.1167/iovs.07-0542 18055803

[B6] Sugars KL Rubinsztein DC Transcriptional abnormalities in Huntington disease Trends Genet 2003 19 5 233 238 10.1016/S0168-9525(03)00074-X 12711212

[B7] Duke DC Moran LB Pearce RK Graeber MB The medial and lateral substantia nigra in Parkinson's disease: mRNA profiles associated with higher brain tissue vulnerability Neurogenetics 2007 8 2 83 94 10.1007/s10048-006-0077-6 17211632

[B8] Ferraiuolo L Heath PR Holden H Kasher P Kirby J Shaw PJ Microarray analysis of the cellular pathways involved in the adaptation to and progression of motor neuron injury in the SOD1 G93A mouse model of familial ALS J Neurosci 2007 27 34 9201 9219 10.1523/JNEUROSCI.1470-07.2007 17715356 PMC6672214

[B9] Chou AH Yeh TH Ouyang P Chen YL Chen SY Wang HL Polyglutamine-expanded ataxin-3 causes cerebellar dysfunction of SCA3 transgenic mice by inducing transcriptional dysregulation Neurobiol Dis 2008 31 1 89 101 10.1016/j.nbd.2008.03.011 18502140

[B10] Blalock EM Geddes JW Chen KC Porter NM Markesbery WR Landfield PW Incipient Alzheimer's disease: microarray correlation analyses reveal major transcriptional and tumor suppressor responses Proc Natl Acad Sci USA 2004 101 7 2173 2178 10.1073/pnas.0308512100 14769913 PMC357071

[B11] Schlamp CL Johnson EC Li Y Morrison JC Nickells RW Changes in Thy1 gene expression associated with damaged retinal ganglion cells Mol Vis 2001 7 192 201 11509915

[B12] Schlamp CL Thliveris AT Li Y Kohl LP Knop C Dietz JA Larsen IV Imesch P Pinto LH Nickells RW Insertion of the beta Geo promoter trap into the Fem1c gene of ROSA3 mice Mol Cell Biol 2004 24 9 3794 3803 10.1128/MCB.24.9.3794-3803.2004 15082774 PMC387761

[B13] Chidlow G Casson R Sobrado-Calvo P Vidal-Sanz M Osborne NN Measurement of retinal injury in the rat after optic nerve transection: an RT-PCR study Mol Vis 2005 11 387 396 15947739

[B14] Levin LA Schlamp CL Spieldoch RL Geszvain KM Nickells RW Identification of the bcl-2 family of genes in the rat retina Invest Ophthalmol Vis Sci 1997 38 12 2545 2553 9375574

[B15] Weishaupt JH Klocker N Bahr M Axotomy-induced early down-regulation of POU-IV class transcription factors Brn-3a and Brn-3b in retinal ganglion cells J Mol Neurosci 2005 26 1 17 25 10.1385/JMN:26:1:017 15968082

[B16] Ivanov D Dvoriantchikova G Nathanson L McKinnon SJ Shestopalov VI Microarray analysis of gene expression in adult retinal ganglion cells FEBS Lett 2006 580 1 331 335 10.1016/j.febslet.2005.12.017 16376886

[B17] Cheng L Sapieha P Kittlerova P Hauswirth WW Di Polo A TrkB gene transfer protects retinal ganglion cells from axotomy-induced death in vivo J Neurosci 2002 22 10 3977 3986 12019317 10.1523/JNEUROSCI.22-10-03977.2002 PMC6757661

[B18] Napankangas U Lindqvist N Lindholm D Hallbook F Rat retinal ganglion cells upregulate the pro-apoptotic BH3-only protein Bim after optic nerve transection Brain Res Mol Brain Res 2003 120 1 30 37 10.1016/j.molbrainres.2003.09.016 14667574

[B19] Park KH Cozier F Ong OC Caprioli J Induction of heat shock protein 72 protects retinal ganglion cells in a rat glaucoma model Invest Ophthalmol Vis Sci 2001 42 7 1522 1530 11381056

[B20] McKinnon SJ Lehman DM Kerrigan-Baumrind LA Merges CA Pease ME Kerrigan DF Ransom NL Tahzib NG Reitsamer HA Levkovitch-Verbin H Quigley HA Zack DJ Caspase activation and amyloid precursor protein cleavage in rat ocular hypertension Invest Ophthalmol Vis Sci 2002 43 4 1077 1087 11923249

[B21] Huang W Fileta J Guo Y Grosskreutz CL Downregulation of Thy1 in retinal ganglion cells in experimental glaucoma Curr Eye Res 2006 31 3 265 271 10.1080/02713680500545671 16531284

[B22] Jenuwein T Allis CD Translating the histone code Science 2001 293 5532 1074 1080 10.1126/science.1063127 11498575

[B23] de la Cruz X Lois S Sanchez-Molina S Martinez-Balbas MA Do protein motifs read the histone code? Bioessays 2005 27 2 164 175 10.1002/bies.20176 15666348

[B24] Shahbazian MD Grunstein M Functions of site-specific histone acetylation and deacetylation Annu Rev Biochem 2007 76 75 100 10.1146/annurev.biochem.76.052705.162114 17362198

[B25] Bottomley MJ Structures of protein domains that create or recognize histone modifications EMBO Rep 2004 5 5 464 469 10.1038/sj.embor.7400146 15184976 PMC1299057

[B26] Lahm A Paolini C Pallaoro M Nardi MC Jones P Neddermann P Sambucini S Bottomley MJ Lo Surdo P Carfi A Koch U De Francesco R Steinkuhler C Gallinari P Unraveling the hidden catalytic activity of vertebrate class IIa histone deacetylases Proc Natl Acad Sci USA 2007 104 44 17335 17340 10.1073/pnas.0706487104 17956988 PMC2077257

[B27] de Ruijter AJ van Gennip AH Caron HN Kemp S van Kuilenburg AB Histone deacetylases (HDACs): characterization of the classical HDAC family Biochem J 2003 370 Pt 3 737 749 10.1042/BJ20021321 12429021 PMC1223209

[B28] Longworth MS Laimins LA Histone deacetylase 3 localizes to the plasma membrane and is a substrate of Src Oncogene 2006 25 32 4495 4500 10.1038/sj.onc.1209473 16532030

[B29] Sengupta N Seto E Regulation of histone deacetylase activities J Cell Biochem 2004 93 1 57 67 10.1002/jcb.20179 15352162

[B30] Li Y Schlamp CL Nickells RW Experimental induction of retinal ganglion cell death in adult mice Invest Ophthalmol Vis Sci 1999 40 5 1004 1008 10102300

[B31] Andreau K Castedo M Perfettini JL Roumier T Pichart E Souquere S Vivet S Larochette N Kroemer G Preapoptotic chromatin condensation upstream of the mitochondrial checkpoint J Biol Chem 2004 279 53 55937 55945 10.1074/jbc.M406411200 15498771

[B32] Drager UC Olsen JF Ganglion cell distribution in the retina of the mouse Invest Ophthalmol Vis Sci 1981 20 3 285 293 6162818

[B33] Li Y Semaan SJ Schlamp CL Nickells RW Dominant inheritance of retinal ganglion cell resistance to optic nerve crush in mice BMC Neurosci 2007 8 19 10.1186/1471-2202-8-19 17338819 PMC1831479

[B34] Quigley HA Nickells RW Kerrigan LA Pease ME Thibault DJ Zack DJ Retinal ganglion cell death in experimental glaucoma and after axotomy occurs by apoptosis Invest Ophthalmol Vis Sci 1995 36 5 774 786 7706025

[B35] Libby RT Li Y Savinova OV Barter J Smith RS Nickells RW John SW Susceptibility to neurodegeneration in a glaucoma is modified by Bax gene dosage PLoS Genet 2005 1 1 17 26 10.1371/journal.pgen.0010004 16103918 PMC1183523

[B36] Li Y Kao GD Garcia BA Shabanowitz J Hunt DF Qin J Phelan C Lazar MA A novel histone deacetylase pathway regulates mitosis by modulating Aurora B kinase activity Genes Dev 2006 20 18 2566 2579 10.1101/gad.1455006 16980585 PMC1578679

[B37] Rouaux C Jokic N Mbebi C Boutillier S Loeffler JP Boutillier AL Critical loss of CBP/p300 histone acetylase activity by caspase-6 during neurodegeneration Embo J 2003 22 24 6537 6549 10.1093/emboj/cdg615 14657026 PMC291810

[B38] Steffan JS Bodai L Pallos J Poelman M McCampbell A Apostol BL Kazantsev A Schmidt E Zhu YZ Greenwald M Kurokawa R Housman DE Jackson GR Marsh JL Thompson LM Histone deacetylase inhibitors arrest polyglutamine-dependent neurodegeneration in Drosophila Nature 2001 413 6857 739 743 10.1038/35099568 11607033

[B39] Klose RJ Zhang Y Regulation of histone methylation by demethylimination and demethylation Nat Rev Mol Cell Biol 2007 8 4 307 318 10.1038/nrm2143 17342184

[B40] Klose RJ Yan Q Tothova Z Yamane K Erdjument-Bromage H Tempst P Gilliland DG Zhang Y Kaelin WG Jr The retinoblastoma binding protein RBP2 is an H3K4 demethylase Cell 2007 128 5 889 900 10.1016/j.cell.2007.02.013 17320163

[B41] Karagianni P Wong J HDAC3: taking the SMRT-N-CoRrect road to repression Oncogene 2007 26 37 5439 5449 10.1038/sj.onc.1210612 17694085

[B42] Boutillier AL Trinh E Loeffler JP Selective E2F-dependent gene transcription is controlled by histone deacetylase activity during neuronal apoptosis J Neurochem 2003 84 4 814 828 10.1046/j.1471-4159.2003.01581.x 12562525

[B43] Karagiannis TC El-Osta A Clinical potential of histone deacetylase inhibitors as stand alone therapeutics and in combination with other chemotherapeutics or radiotherapy for cancer Epigenetics 2006 1 3 121 126 17965606 10.4161/epi.1.3.3328

[B44] Jiang H Nucifora FC Jr Ross CA DeFranco DB Cell death triggered by polyglutamine-expanded huntingtin in a neuronal cell line is associated with degradation of CREB-binding protein Hum Mol Genet 2003 12 1 1 12 10.1093/hmg/ddg002 12490527

[B45] Jin K Mao XO Simon RP Greenberg DA Cyclic AMP response element binding protein (CREB) and CREB binding protein (CBP) in global cerebral ischemia J Mol Neurosci 2001 16 1 49 56 10.1385/JMN:16:1:49 11345520

[B46] McCampbell A Taye AA Whitty L Penney E Steffan JS Fischbeck KH Histone deacetylase inhibitors reduce polyglutamine toxicity Proc Natl Acad Sci USA 2001 98 26 15179 15184 10.1073/pnas.261400698 11742087 PMC65003

[B47] Pallos J Bodai L Lukacsovich T Purcell JM Steffan JS Thompson LM Marsh JL Inhibition of specific HDACs and sirtuins suppresses pathogenesis in a Drosophila model of Huntington's disease Hum Mol Genet 2008 17 23 3767 3775 10.1093/hmg/ddn273 18762557 PMC2581431

[B48] Hockly E Richon VM Woodman B Smith DL Zhou X Rosa E Sathasivam K Ghazi-Noori S Mahal A Lowden PA Steffah JS Marsh JL Thompson LM Lewis CM Marks PA Bates GP Suberoylanilide hydroxamic acid, a histone deacetylase inhibitor, ameliorates motor deficits in a mouse model of Huntington's disease Proc Natl Acad Sci USA 2003 100 4 2041 2046 10.1073/pnas.0437870100 12576549 PMC149955

[B49] Saha RN Pahan K HATs and HDACs in neurodegeneration: a tale of disconcerted acetylation homeostasis Cell Death Differ 2006 13 4 539 550 10.1038/sj.cdd.4401769 16167067 PMC1963416

[B50] Ryu H Lee J Olofsson BA Mwidau A Dedeoglu A Escudero M Flemington E Azizkhan-Clifford J Ferrante RJ Ratan RR Histone deacetylase inhibitors prevent oxidative neuronal death independent of expanded polyglutamine repeats via an Sp1-dependent pathway Proc Natl Acad Sci USA 2003 100 7 4281 4286 10.1073/pnas.0737363100 12640146 PMC153084

[B51] Chen B Cepko CL HDAC4 regulates neuronal survival in normal and diseased retina Science 2009 323 5911 256 259 10.1126/science.1166226 19131628 PMC3339762

[B52] Seo HW Kim EJ Na H Lee MO Transcriptional activation of hypoxia-inducible factor-1alpha by HDAC4 and HDAC5 involves differential recruitment of p300 and FIH-1 FEBS Lett 2009 583 1 55 60 10.1016/j.febslet.2008.11.044 19071119

[B53] Weber AJ Harman CD BDNF preserves the dendritic morphology of alpha and beta ganglion cells in the cat retina after optic nerve injury Invest Ophthalmol Vis Sci 2008 49 6 2456 2463 10.1167/iovs.07-1325 18263808

[B54] Li Y Schlamp CL Poulsen KP Nickells RW Bax-dependent and independent pathways of retinal ganglion cell death induced by different damaging stimuli Exp Eye Res 2000 71 2 209 213 10.1006/exer.2000.0873 10930325

[B55] Bortner CD Sifre MI Cidlowski JA Cationic gradient reversal and cytoskeleton-independent volume regulatory pathways define an early stage of apoptosis J Biol Chem 2008 283 7219 7229 10.1074/jbc.M707809200 18187415 PMC2680553

[B56] Redman PT He K Hartnett KA Jefferson BS Hu L Rosenberg PA Levitan ES Aizenman E Apoptotic surge of potassium currents is mediated by p38 phosphorylation of Kv2.1 Proc Natl Acad Sci USA 2007 104 3568 3573 10.1073/pnas.0610159104 PMC1805571 17360683

[B57] Koeberle PD Wang Y Schlichter LC Kv1.1 and Kv1.3 channels contribute to the degeneration of retinal ganglion cells after optic nerve transection in vivo Cell Death Differ 2010 17 134 44 10.1038/cdd.2009.113 19696788

[B58] Azarian SM Schlamp CL Williams DS Characterization of calpain II in the retina and photoreceptor outer segments J Cell Sci 1993 105 Pt 3 787 798 8408304 10.1242/jcs.105.3.787

[B59] Pelzel HR Schlamp CL Poulsen GL Ver Hoeve JA Nork TM Nickells RW Decrease of cone opsin mRNA in experimental ocular hypertension Mol Vis 2006 12 1272 1282 17110910

[B60] Schlamp CL Nickells RW Light and dark cause a shift in the spatial expression of a neuropeptide-processing enzyme in the rat retina J Neurosci 1996 16 7 2164 2171 8601797 10.1523/JNEUROSCI.16-07-02164.1996 PMC6578520

[B61] Blanchard F Chipoy C Histone deacetylase inhibitors: new drugs for the treatment of inflammatory diseases? Drug Discov Today 2005 10 3 197 204 10.1016/S1359-6446(04)03309-4 15708534

[B62] Khan N Jeffers M Kumar S Hackett C Boldog F Khramtsov N Qian X Mills E Berghs SC Carey N Finn PW Collins LS Tumber A Ritchie JW Jensen PB Lichenstein HS Sehested M Determination of the class and isoform selectivity of small-molecule histone deacetylase inhibitors Biochem J 2008 409 2 581 589 10.1042/BJ20070779 17868033

