# Supplementary figures and images for: Histone H4 deacetylation plays a critical role in early gene silencing during neuronal apoptosis
Source: BMC Neurosci. 2010 May 26;11:62. doi: 10.1186/1471-2202-11-62 (PMC2886060; doi:10.1186/1471-2202-11-62)

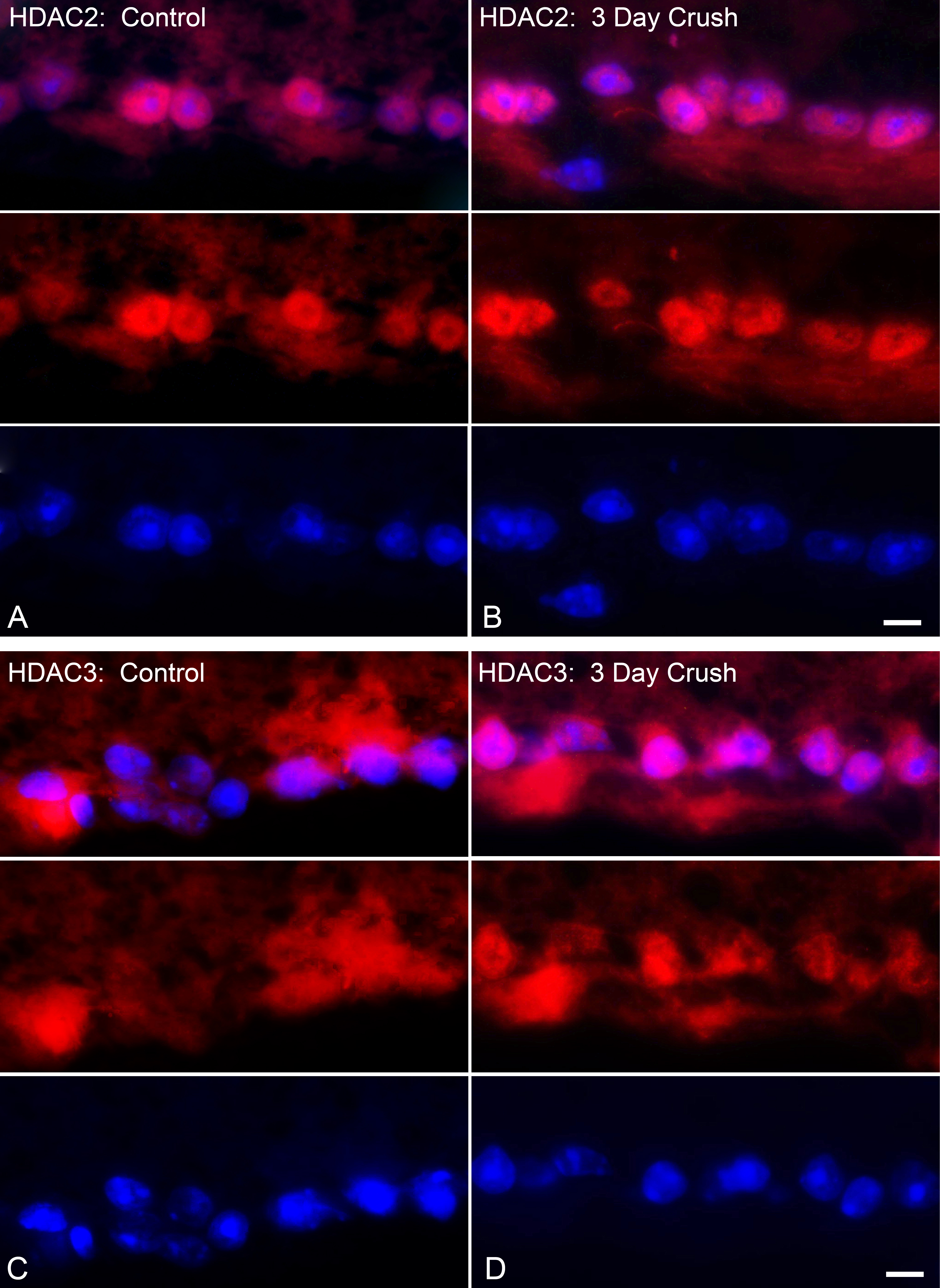

Supplement: Additional file 1 — Localization of HDACs 2 and 3 before and after optic nerve crush. Photomicrographs of the individual channels for the merged images of Figure 3B. Sections from control retinas and retinas 3 days after optic nerve crush (ONC) were labeled with antibodies against HDAC2 or HDAC3 and counter-stained with DAPI to highlight nuclei. High-magnification images of the GCL are shown. (A, B) In control and crush retinas, HDAC2 labeling (red) was present in the nuclei of cells in the GCL, as determined by DAPI staining (blue). (C) In control retinas, HDAC3 labeling had a diffuse, cytoplasmic appearance with minimal overlap with nuclear DAPI staining. (D) At 3 days post ONC, many cells in the GCL exhibited nuclear HDAC3 labeling that coincided with DAPI staining of the nuclei. Scale bar = 5 μm. [file 1471-2202-11-62-S1.JPEG]

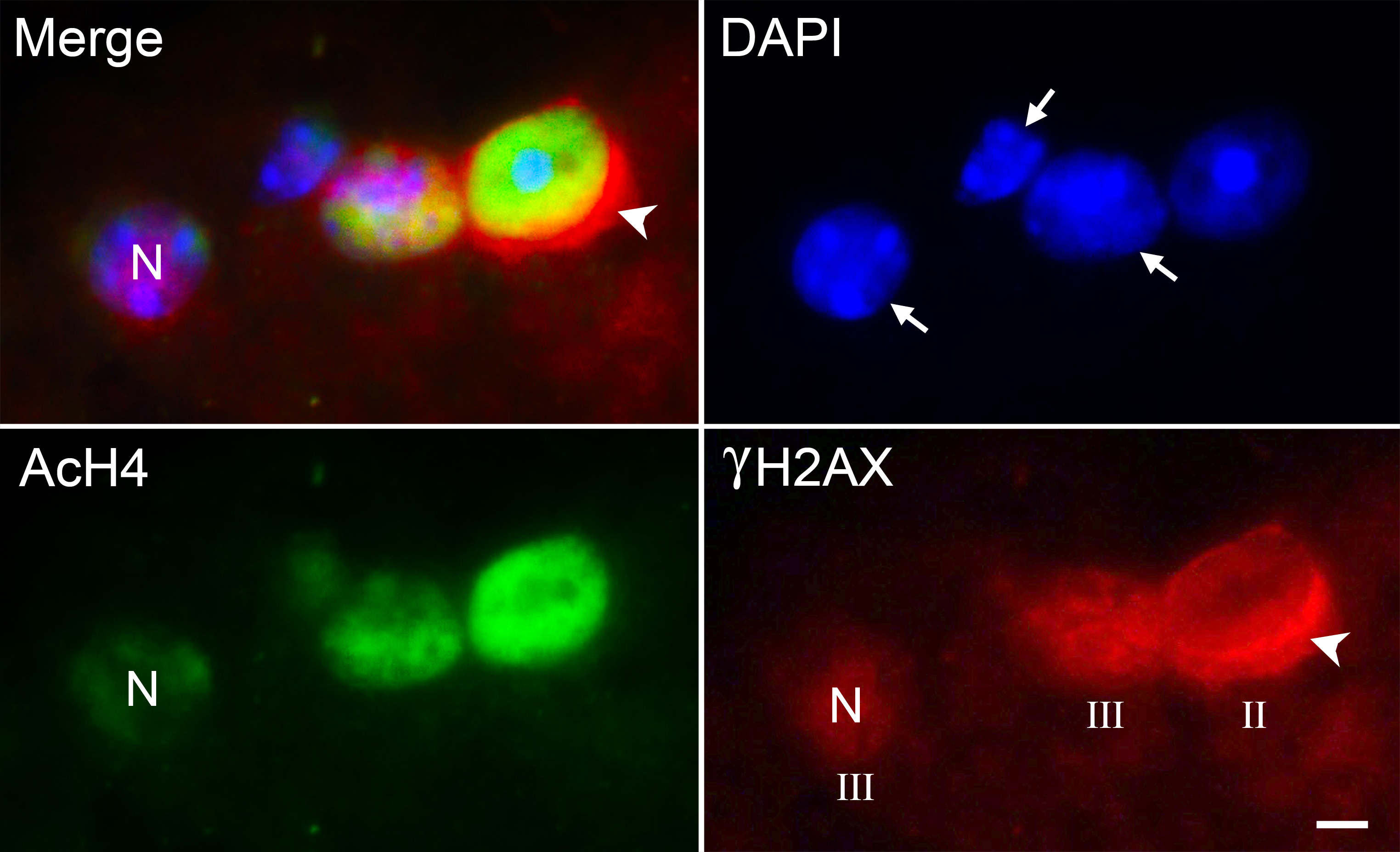

Supplement: Additional file 2 — The deacetylation of histone H4 occurs in cells with nuclear γH2AX and DNA fragmentation. Sections from a retina after optic nerve injury were double labeled with antibodies against acetylated histone H4 (AcH4, green) and γH2AX (red), to identify apoptotic cells, and counter-stained with DAPI (blue) to verify the nuclear presence of the proteins. Four cells in the ganglion cell layer are visible in various stages of cell death. DAPI-staining of 3 of the cells show condensed and fragmenting nuclei, consistent with apoptosis (arrows). One cell exhibits a normal nuclear staining pattern, including the presence of a robust nucleolus. Two of the cells with condensed chromatin also exhibit stage III γH2AX staining and all 3 cells are weakly staining for AcH4. The relatively normal appearing cell exhibits both strong stage II γH2AX (arrowhead) and AcH4 staining. A single representative nucleus, showing of DNA fragmentation and stage III γH2AX labeling is indicated (N). Size bar = 5 μm. [file 1471-2202-11-62-S2.JPEG]

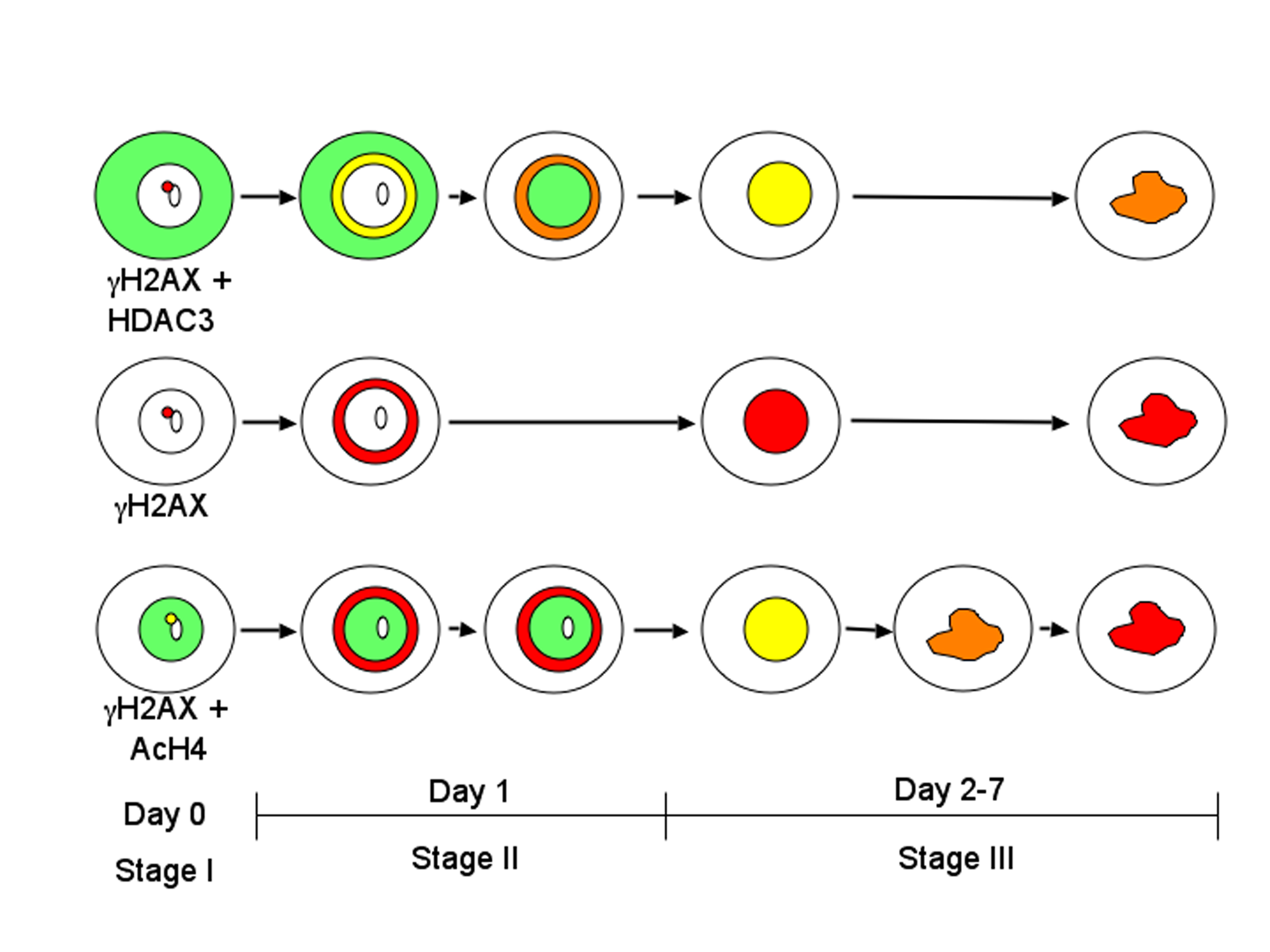

Supplement: Additional file 3 — The change in expression and cellular distribution of γH2AX provides a temporal indicator of histone deacetylation in damaged ganglion cells. A graphic representation of the co-localization of γH2AX staining and HDAC3 or Acetyl H4 (AcH4), indicates that HDAC3 is translocated into nuclei of apoptotic cells before the loss of AcH4 staining. The top row of cells represents the co-localization of γH2AX with HDAC3, while the bottom row represents the co-localization of γH2AX with AcH4. The labeling pattern of just γH2AX is shown in the center row of cells. In each row, γH2AX staining is depicted in red, while HDAC3 and AcH4 are depicted in green. At day 0, cells are negative for γH2AX staining, with the exception of a spot adjacent to the nucleolus. These cells are classified as stage I, and exhibit cytosolic staining for HDAC3 and nuclear staining for AcH4. Once apoptosis is initiated, γH2AX-staining appears as a perinuclear ring (stage II, principally detected at day 1 after optic nerve crush), which appears yellow in some cells co-stained for HDAC3 (Figures 6B and 12C), indicating complete co-localization. Some cells, however, appear to have a more orange ring with a green nucleus, suggesting movement of HDAC3 from the cytosol to the nucleus in stage II cells (Figures 6B asterisk, 6C, 12E). In cells with nuclear γH2AX-staining (stage III, detected in increasing amounts at 3, 5, and 7 days post optic nerve crush), cells co-labeled for HDAC3 exhibit yellow to orange nuclei, indicating co-localization of these proteins (Figures 6D-F, 12H, 12J). With respect to AcH4 labeling, stage I cells exhibit bright green nuclei. Stage II cells typically appear as red rings of γH2AX label surrounding green nuclei (Figures 7B, 12C). Stage III cells exhibit three different nuclear colors, indicative of the extent of AcH4 label present, and appear to progress from yellow (Figures 7D, 12F), to orange (Figures 7E, 12G), to red (Figures 7F, 12I). Orange to red nuclei are often misshapen, a [file 1471-2202-11-62-S3.JPEG]
